# Amorphous Solid Dispersions: Implication of Method of Preparation and Physicochemical Properties of API and Excipients

**DOI:** 10.3390/pharmaceutics16081035

**Published:** 2024-08-02

**Authors:** Varun Kushwah, Cecilia Succhielli, Isha Saraf, Amrit Paudel

**Affiliations:** 1 Research Center Pharmaceutical Engineering GmbH, 8010 Graz, Austriaisha.saraf@rcpe.at (I.S.); 2 Institute of Pharmaceutical Science, Department of Pharmaceutical Technology, University of Graz, 8010 Graz, Austria

**Keywords:** amorphous solid dispersions, hot melt extrusion, spray drying, carvedilol, ibuprofen, fenofibrate, Soluplus^®^, hydroxypropyl methylcellulose

## Abstract

The present study investigated the effect of different polymers and manufacturing methods (hot melt extrusion, HME, and spray drying, SD) on the solid state, stability and pharmaceutical performance of amorphous solid dispersions. In the present manuscript, a combination of different binary amorphous solid dispersions containing 20% and 30% of drug loadings were prepared using SD and HME. The developed solid-state properties of the dispersions were evaluated using small- and wide-angle X-ray scattering (WAXS) and modulated differential scanning calorimetry (mDSC). The molecular interaction between the active pharmaceutical ingredients (APIs) and polymers were investigated via infrared (IR) and Raman spectroscopy. The in vitro release profile of the solid dispersions was also evaluated to compare the rate and extend of drug dissolution as a function of method of preparation. Thereafter, the effect of accelerated stability conditions on the physicochemical properties of the solid dispersions were also evaluated. The results demonstrated higher stability of Soluplus^®^ (SOL) polymer-based solid dispersions as compared to hydroxypropyl methylcellulose (HPMC)-based solid dispersions. Moreover, the stability of the solid dispersions was found to be higher in the case of API having high glass transition temperature (Tg) and demonstrated higher interaction with the polymeric groups. Interestingly, the stability of the melt-extruded dispersions was found to be slightly higher as compared to the SD formulations. However, the down-processing of melt-extruded strands plays critical role in inducing the API crystal nuclei formation. In summary, the findings strongly indicate that the particulate properties significantly influence the performance of the product.

## 1. Introduction

Oral solid dosage is the preferred dosage form owing to its relative ease of production, high physicochemical stability and patient compliance. However, the efficacy of oral administration of poorly water-soluble drugs (BSC class II and IV) was limited due to reduced gastric bioavailability [1]. Among various drug delivery platforms, amorphous solid dispersions are one of the most successful strategies to enhance the solubility and dissolution rate in poorly water-soluble drugs [2,3].

An amorphous solid dispersion (ASD) can be defined as a dispersion of one or more active ingredients (API) in hydrophilic carriers [4]. The API molecules in the dispersion exist in amorphous solid state, homogenously mixed with the carrier molecules at the molecular level. The ultimate goal of development of amorphous molecular dispersion is to ameliorate the API release rate and thereafter maintain the supersaturation state under in vivo conditions, leading to higher API permeability across the intestinal wall [5,6].

Among various method of preparation of pharmaceutical ASDs, hot melt extrusion (HME) and spray drying (SD) are the two most common industrial manufacturing process [7,8,9]. In spite of a number of ASD products paved their way to market, very limited information is available, evaluating the impact of different methods and excipient types on product performance, in terms of stability and bioavailability of the API [8,9,10,11]. Owing to different mechanisms involved (mainly driven by selected method and excipient) the API in the developed formulation could exist in different high energy states, resulting in varied chemical and physical stability during shelf life period.

Thus, in accordance with filling the void, a thorough investigation with a view to understanding the influence of different manufacturing processes and types of excipient on the success rate and physicochemical properties of the solid dispersion is required during the early-stage development of products. The present study compared the physicochemical properties and in vitro dissolution performances of ASDs prepared via two standard manufacturing processes, namely, HME followed by cryogenic milling and SD. Moreover, the present study demonstrated the impact of manufacturing process on the physical structure, stability and pharmaceutical performance of the API.

Three BCS class II drugs, i.e., carvedilol (CAR), ibuprofen (IBU) and fenofibrate (FB), were investigated having different chemical structures (functional groups) with different propensities of physicochemical interactions (with carriers) and diverse glass transition temperature (Tg) values. Among them, IBU (–COOH), CAR (–OH) and FB (no specific H–donor group) represent APIs with strong, weak and poor H-bonding tendency, respectively. CAR, FB and IBU exhibit 39.6 °C (highest), −20.0 °C (medium) and −45.3 °C (lowest) Tg values, respectively.

Soluplus^®^ (SOL) and hydroxypropyl methylcellulose (HPMC), amorphous and hydrophilic polymeric ASD carriers, were used for the development of solid dispersion. SOL and HPMC possess diverse physicochemical properties, with higher thermoplasticity, and a wide range of solubility of SOL in class II and III solvents as compared to HPMC, whereas HPMC possess ether and alcohol groups, which are relatively weaker H-bond acceptors. Moreover, SOL comprises H acceptor (acetate and caprolactam), ether and alcohol (relatively weaker H-bond acceptors) groups, which are prerequisite for the stabilization of intermolecular interaction with API. The developed solid dispersions were then characterized for the solid state, physicochemical properties and API–polymer interactions, and compared to evaluate the effect of preparation methods (SD and HME), and excipients (HPMC and SOL) on stabilizing the API (with diverse properties) in the amorphous solid state. The properties of the investigated APIs and excipients are mentioned in Table 1.

## 2. Material and Methods

### 2.1. Material

Carvedilol (CAR), ibuprofen (IBU) and fenofibrate (FB) were purchased from Indoco Remedies (Mumbai, India), Shin-Etsu Chemical Co., Ltd. (Tokyo, Japan) and Haihang Industry Co., Ltd. (Jinan, China), respectively, and were as per USP quality. Polyvinyl caprolactam–polyvinyl acetate–polyethylene glycol graft copolymer (PCL-PVAc-PEG) with the polymerization ratio of 57:30:13 [21], commonly known as Soluplus^®^ (SOL), was obtained from BASF SE (Ludwigshafen, Germany). Hydroxypropyl methylcellulose (HPMC) of extrusion grade (AFFINISOL™ 100 LV), a semisynthetic inert viscoelastic polymer, was procured from Dow Chemical (Dow Chemical Company, Stade, Germany). Ethanol (purity > 99.9%) was purchased from Merck KGaA (Darmstadt, Germany). Sodium lauryl sulphate (SLS) used within the dissolution studies was purchased from Carl Roth^®^ (Karlsruhe, Germany). Sodium phosphate dibasic dodecahydrate and potassium phosphate monobasic, used for preparing the phosphate buffer solution, were purchased from Sigma Aldrich^®^ (Stenheim, Germany). Potassium chloride, the salt used for preparing the hydrochloric acid buffer solution, was purchased from Carl Roth^®^ (Karlsruhe, Germany), while the hydrochloric acid 37% was purchased from Sigma Aldrich^®^ (Stenheim, Germany).

### 2.2. Methods

Table 2 summarizes the combination of API and polymer for the preparation of binary ASDs via spray drying and hot melt extrusion.

#### 2.2.1. Hot Melt Extrusion

Hot melt extrusion (HME) experiments were carried out using a co-rotating twin-screw Three Tec Table Top Mini Extruder ZE 9 (Seon, Switzerland) with a 1.5 mm orifice die. Extrusion blends with 20% and 30% of drugs concentrations were prepared by mixing separately with SOL and HPMC polymers for 30 min using a shake mixer Turbula^®^ T2F (WAB) at 60 rpm. The mixture blend was manually fed into the extruder (with temperature 115 °C and screw speed at 60 rpm) and the extrudates were collected as strands.

The collected extrudates were then cryo-milled using Retsch CryoMill (Haan, Germany) equipped with stainless steel ball. The extrudate samples were pre-cooled in cryomill (−196 °C, with liquid nitrogen) at a milling frequency of 5 Hz, followed by milling at 25 Hz for 3 cycles. The duration of cycles was kept constant with 2.30 min and the time interval between each cycle was kept at 1 min. with a frequency of 5 Hz. Post cryo-milling, the samples were stored in desiccator at 4 ± 1 °C.

#### 2.2.2. Spray Drying

Amorphous solid dispersions made up of 20% *w*/*w* and 30% *w*/*w* of APIs in SOL were prepared by spray-drying 5% *w*/*v* solutions of drug and polymer in ethanol. The solutions were spray-dried using a Nano Spray Dryer B-90 (Buchi, Switzerland) (with an inlet temperature of 80 °C, 7 µm atomization mesh, and spray rate of 0.41 ± 0.17 mL/min). The drying gas flow rate was fixed to 110 L/min. The spray-dried samples were further vacuum dried to remove the residual solvent and stored in a desiccator at 4 ± 1 °C.

#### 2.2.3. FTIR Spectroscopy

Solid-state interaction between drug and polymer in solid dispersions was evaluated using attenuated total internal reflection (ATR) FTIR (VERTEX 70, Bruker Optik GmbH, Ettlingen, Germany). The spectra were collected as the average of 64 scans acquired in the spectral range from 600 to 4500 cm^−1^ with a spectral resolution of 4 cm^−1^ in triplicate. The IR spectra were absorbance normalized with respect to the most intense peak. Digital physical mixtures were derived by combining the weighted individual spectra of pure API and polymer. IR spectral data analysis was performed using Spekwin32 optical spectroscopy software (V. 1.71.6.1).

#### 2.2.4. Raman Spectroscopy

The samples (raw materials, spray-dried samples, extrudate samples and stability samples) were also analyzed for evaluation of drug–polymer chemical interaction using the dispersive Raman spectrometer (Raman Station 400, PerkinElmer, Waltham, MA, USA) in triplicate. The sample was irradiated with a 785 nm near-infrared diode laser (laser power 100 mW, aperture of 50 µm). Using a super macro point sampling, 7 scans were taken (with an exposure time of 5 s for each scan) in the spectral rage from 400 to 3500 cm^−1^ and a spectral resolution of 2 cm^−1^. Likewise, for IR spectra, all Raman spectra were also *y*-axis-normalized with respect to the highest intensity. Digital physical mixtures were derived by combining the weighted individual spectra of pure API and polymer. All Raman spectra were analyzed using Spekwin32 optical spectroscopy software (V. 1.71.6.1).

#### 2.2.5. Differential Scanning Calorimetry (DSC)

The modulated DSC (mDSC) analysis of samples were performed using DSC 204 F1 Phönix (Netzsch, Germany) in a dry nitrogen purged chamber at a flow rate of 50 mL/min. All samples were analyzed in duplicate using 40 μL aluminum pans (samples weighed and crimped with the manually pierced aluminum lid). The heating range of −60 °C to 130 °C, 0 °C to 130 °C and −40 °C to 130 °C was applied for the IBU, CAR and FB systems (solid dispersion with both SOL and HPMC polymers), respectively. The samples were heated from at a rate, amplitude and frequency of 2 °C/min, ±1 °C and 60 s, respectively. The DSC data were analyzed using NETZSCH Proteus Thermal Analysis V.6.1.0 with SW-TMDSC-69X.1B.

#### 2.2.6. X-ray Scattering Study

Simultaneous small- and wide-angle X-ray scattering (SWAXS) analyses were performed to determine the solid-state crystallinity in the samples using S3-MICRO camera (Bruker AXS, Karlsruhe, Germany) at 20 ± 1 °C. The samples were held in quartz capillaries with a length of 80 mm, an outside diameter of 2 ± 0.1 mm and a wall thickness of 0.01 mm. WAXS measurements were performed in the angular range of 17–27° 2 θ, which corresponds to the real space dimensions of 4.9 and 3.3 Å. The exposure duration for measurement was 600 s. The SWAXS data was analyzed using 3D-View software version 1.

#### 2.2.7. In Vitro Drug Release

Dissolution studies of the 20% drug loaded extrudate and spray-dried samples were performed using United State Pharmacopoeia type II dissolution test apparatus (paddle) (Erweka DT820LH Dissolution Tester, Heusenstamm, Germany) at 37 ± 0.5 °C. Briefly, samples were transferred into the dissolution vessel containing 500 mL of phosphate buffer (pH 6.8) with 0.1% *w*/*v* SLS as a dissolution media. For the dissolution of IBU loaded systems, formulations equivalent to 36 mg of API were added in the dissolution medium of hydrochloric acid (HCl) solution (pH 1.2) with 0.1% *w*/*v* SLS. For CAR-loaded formulations, samples equivalent to 16 mg of API were added in the phosphate buffer (pH 6.8) with 0.1% *w*/*v* SLS. In the case of FB-loaded solid dispersions, formulations equivalent to 10 mg of API were added in the HCl solution (pH 1.2) with 0.2% *w*/*v* SLS.

Dissolution testing was performed using a paddle rotation speed of 100 rpm. Then, 1.5 mL of filtered sample, using a 10 μm membrane filter (10 μm Poroplast Filters, Erweka), was automatically withdrawn in amber glass vials at 5, 10, 15, 20, 30, 40 and 60 min and replaced with the fresh dissolution media. Thereafter, 0.5 mL of the samples were diluted with equivalent amount of ethanol and analyzed using HPLC methods (please find the details in Supplementary Information).

In order to nullify the effect to different particle size of on the dissolution profile, the cryo-milled extrudate powders were sieved using a Retsch^®^ vibratory sieve Shaker AS 200 control (Haan, Germany). The sieving was carried out over 15 min, with amplitude of 1.00 mm/g. The cryo-milled particles were screened using three mesh sizes of 45, 90 and 200 μm, and the sample fraction with particle size of range 45 to 90 μm was used for the dissolution studies.

#### 2.2.8. Stability Studies

Approx. 200 mg of the samples were kept at stability condition of 40 °C and 75%RH (KBF 240, Binder, Tuttlingen, Germany) and were analyzed after 7, 14 and 21 days using infrared spectroscopy, Raman spectroscopy, WAXS and mDSC, as mentioned above.

## 3. Results and Discussion

### 3.1. Physicochemical Characterization

#### 3.1.1. CAR-Loaded Solid Dispersion

##### FTIR Spectroscopy

**Figure 1:**
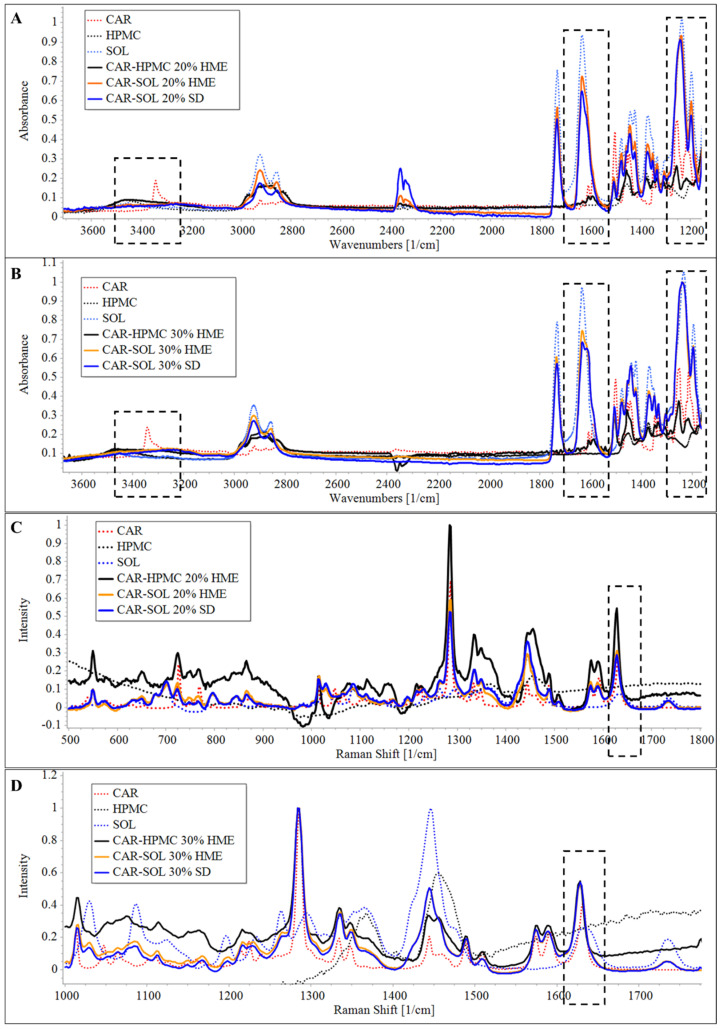
FTIR (**A**,**B**) and Raman spectra (**C**,**D**) of 20% and 30% CAR loaded spray-dried and melt-extruded dispersions, respectively.

As is evident from Figure 1A,B, the FTIR spectrum of crystalline CAR demonstrated a characteristic IR absorption band at 3342.5 cm^−1^, corresponding to the overlapping of the secondary amine NH stretching vibration and to the OH stretching vibration [15,22]. The amorphous CAR exhibits acharacteristic broad peak at 3398.8 cm^−1^, instead of a sharp peak at 3342.5 cm^−1^ (Figure S1).

To evaluate the polymer’s capacity to maintain the amorphous structure, a solid dispersion of CAR with SOL and HPMC was formulated via HME and characterized. The FTIR spectra of melt-extruded SOL- and HPMC-based solid dispersions containing 20% and 30% of CAR demonstrated absence of characteristic peak of CAR at 3342.5 cm^−1^. Furthermore, a broad peak at 3398.8 cm^−1^ was observed in the case of both solid dispersions, suggesting the presence of CAR in amorphous structures.

In the case of CAR-HPMC extrudates, broadening of HPMC and CAR signals at wavelength 1150–950 cm^−1^ corresponding to the C–O stretching vibration of ethereal and hydroxyl groups, respectively, which could be due to higher drug–polymer interaction. However, a redshift in the C–O stretching peak of CAR was found, suggesting weak H-bonding between C–O (CAR) and OH (HPMC) groups. The FTIR spectra of CAR-SOL extrudate demonstrated a blueshift in the SOL acetate C–O stretching, indicating presence of strong H-bonding in CAR-SOL (between C–O group of SOL and OH/NH group of CAR) as compared to CAR-HPMC solid dispersion system.

Thereafter, the influence of the HME and SD processes on solid dispersion performance was also evaluated. In both HME and SD dispersions containing 30% drug content the acetate C–O peak of SOL shifted from 1233 cm^−1^ to 1239 cm^−1^. In the dispersions containing 20% drug content, the peak maxima shifted to 1240 cm^−1^ and 1236 cm^−1^ for the SD and HME dispersion, respectively. The shift in peak maxima could be due to the higher polymer chain opening and subsequent incorporation of API molecules within the inter-chains, leading to decreased polymer–polymer (dipolar) interaction. The peaks attributed to C=O stretching of SOL acetate group (1732 cm^−1^) did not demonstrate any peak shift. However, it is apparent in the spectra of dispersions that the peak maxima of the large fraction of caprolactam C=O remained as of pure polymer at 1633 cm^−1^, while a clear low wavenumber shoulder is visible in the spectra of all ASDs. The latter peak (1611 cm^−1^) could be attributable to the caprolactam C=O fractions that are H-bonded with the OH of CAR. The relative intensity of the peak at 1611 cm^−1^ is higher for the higher drug loading, while slightly higher intensity and peak shoulder was found in the case of SD method preparation as compared to HME, suggesting stronger interactions for CAR loaded SD formulations.

In addition to the peak shift, broadening of the CAR peak was also observed, demonstrating formation of amorphous structures of API. The peak broadening could be due to disruptions of long-range order upon amorphization, majorly accompanied by the alterations to intermolecular arrangements in NH and OH moieties that are otherwise predominantly involved in inter- and intramolecular H-bonding in the crystalline state [15].

##### Raman Spectroscopy

As is evident from Figure 1C,D, the spectra of crystalline CAR demonstrated characteristic peaks at 914 cm^−1^ and 540 cm^−1^ attributed to CCC/CNC (carbazole) and CH_2_ rocking (linear chain) deformation and a strong band at 1633 cm^−1^ corresponding to C–C stretching (carbazole) [23,24].

In the case of both HPMC and SOL solid dispersion systems, a peak shift from 1633 cm^−1^ to 1628 cm^−1^ was observed, which could be due to the molecular dispersion of CAR in the ASDs (zoomed Raman spectra are provided in supplementary information sheet, Figure S6).

Moreover, in the SOL-based solid dispersion, the contribution of free caprolactam peak of SOL was found to significantly less as compared to free SOL. This signifies the presence of the predominant fraction of caprolactam C=O (at 1634 cm^−1^) involved in H-bonding with the OH of CAR; the results were in accordance with the IR.

##### mDSC

**Figure 2:**
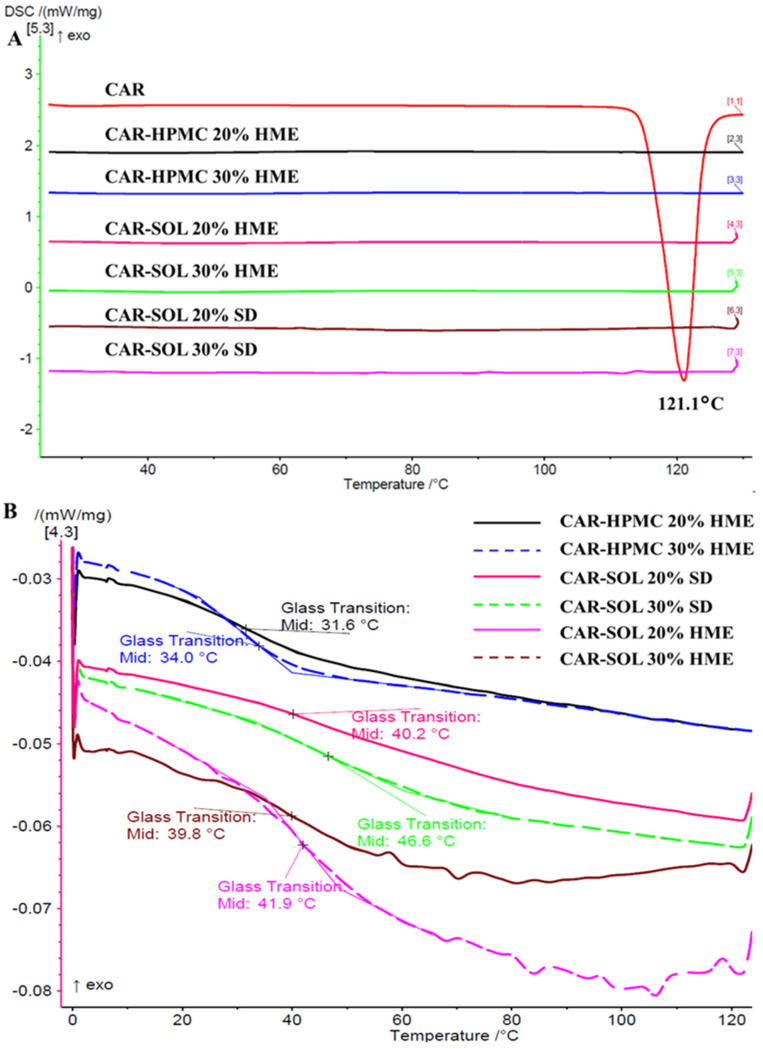
(**A**) Total and (**B**) reversing (showing Tg) mDSC thermograms of 20% and 30% CAR loaded solid dispersions.

As shown in Figure 2, the mDSC thermograms of 20% and 30% CAR-SOL- and CAR-HPMC-based solid dispersion demonstrated no melting endotherm at 121.1 °C. Furthermore, all the mDSC thermograms of melt-extruded and spray-dried solid dispersions demonstrated presence of single broad glass transition event, indicating presence of amorphous structures in all the formulations. The mDSC curves of the raw materials provided in the supplementary information sheet (Figures S3–S5).

##### WAXS

The WAXS profile of crystalline CAR demonstrated characteristic peaks at 2θ values 18°, 19°, 20°, 21°, 22.6°, 24° and 26°. The developed CAR-SOL and HPMC solid dispersions were also analyzed for the evaluation of crystalline fraction of API in the system. As evident from the Figure S2, no characteristic Bragg peaks of CAR were found in the solid dispersions prepared via SD and HME, indicating the dispersion of drug particles at molecular level in the polymer matrices.

#### 3.1.2. IBU-Loaded Solid Dispersion

##### FTIR Spectroscopy

**Figure 3:**
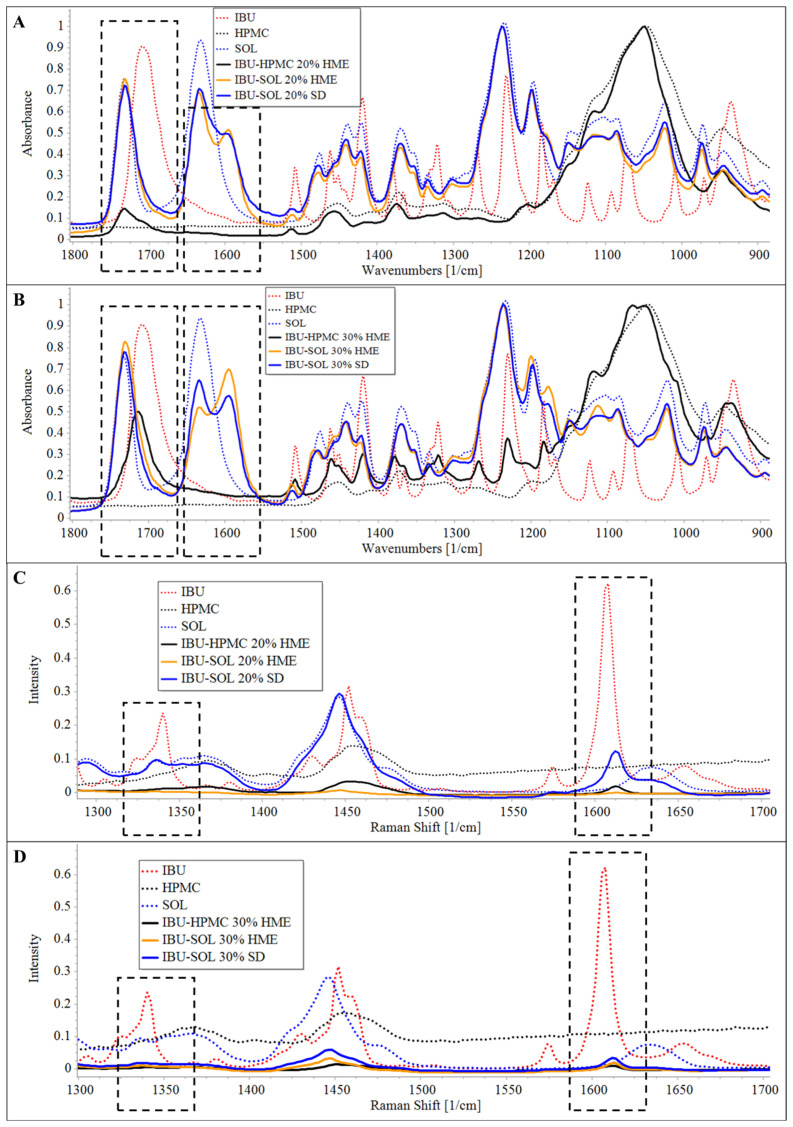
FTIR (**A**,**B**) and Raman spectra (**C**,**D**) of 20% and 30% IBU loaded spray-dried and melt-extruded dispersions, respectively.

For the HPMC-based solid dispersion, the peak maxima of 1708 cm^−1^ (carbonyl group) was found to be shifted to 1732 cm^−1^ and 1715 cm^−1^ in the case of 20% and 30% loading, respectively (Figure 3A,B). The peak shift to 1715 cm^−1^, demonstrated amorphous composites of IBU with some level of heterogeneity with HPMC in extrusion. In addition to the peak maxima of 1732 cm^−1^, the 20% drug-loaded formulation also demonstrated low-intensity signals at 1715 cm^−1^.

The signals at 1708 cm^−1^ corresponding to the C=O stretching of the carboxylic acid of IBU, which are H-bonded to the OH molecule of another IBU molecules to form a cyclic dimer in crystalline structures [25,26]. Shift in 1708 cm^−1^ peak to the higher wavenumber in the solid dispersions, suggests the formation of considerable fraction of IBU monomer by the breakage of IBU-IBU H-bonds, leading to amorphous structures of IBU.

In the case of both 20 and 30% IBU-loaded SOL melt-extruded solid dispersion, the peak maxima were shifted from 1708 cm^−1^ to 1732 cm^−1^. Thus, in line with the CAR solid dispersion, SOL demonstrated higher potential in development of amorphous solid dispersion of IBU, as compared to HPMC.

Subsequently, the IBU-SOL solid dispersion was also prepared using SD and compared with the melt-extruded formulation. In both SD and HME formulations, the peak intensity of free acetate C=O and caprolactam C=O groups were found to be comparable and significantly decreased, respectively, as compared to pure SOL. In addition to the free caprolactam C=O peak, a new distinct signal with peak maxima 1595 cm^−1^ was also observed in all solid dispersions, which could be attributable to the vibration band of the caprolactam C=O fraction of SOL, H-bonded with the OH group of IBU.

Moreover, the ratio of H-bonded (1595 cm^−1^) to free (1633 cm^−1^) caprolactam C=O bands was found to be dependent on drug loading and the method of preparation. For 20% drug loading, the peak intensity ratios of 1595 cm^−1^ to 1633 cm^−1^ was found to be 0.66 and 0.74 in the case of SD and HME samples, respectively. For solid dispersions with 30% drug loading, the peak intensity ratios of 1595 cm^−1^ to 1633 cm^−1^ were found to be 0.88 and 1.33 for SD and HME samples, respectively. The results indicated higher fraction of H-bonded caprolactam for HME dispersions than that in the SD counterparts. These findings evidently suggest that API as well as polymer molecules possibly follow diverse molecular manifestations while experiencing different processes of amorphous dispersion mixing.

##### Raman Spectroscopy

As shown in Figure 3C,D, the Raman band of crystalline IBU shows characteristic peak maxima at 1608 cm^−1^, assigned to vibrations of aryl ring C=C bonds [27,28]. The Raman spectra of HPMC- and SOL-based solid dispersions showed peak shifts from 1608 cm^−1^ (pure crystalline IBU) to 1612 cm^−1^ (zoomed Raman spectra are provided in Supplementary Information, Figure S9). The shift in the peak is attributed to the glassy amorphous state of the IBU in the dispersions; the results were in agreement with previous studies reported by Hédoux et al. [29].

##### mDSC

**Figure 4:**
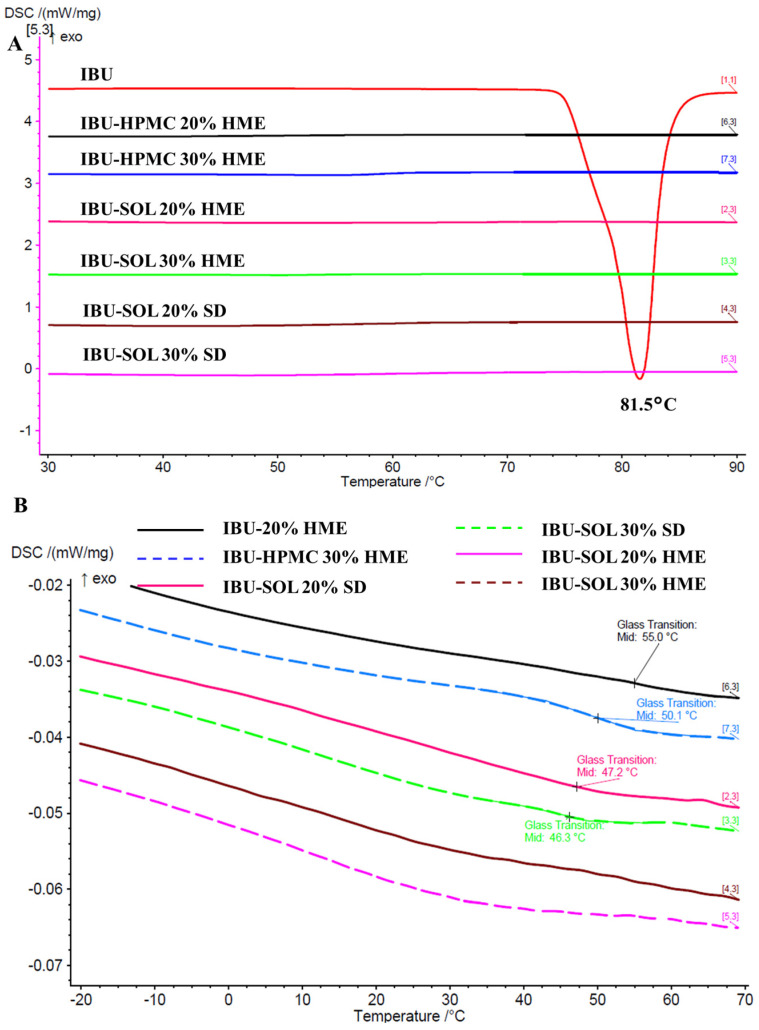
(**A**) Total and (**B**) reversing (showing Tg) mDSC thermograms of 20% and 30% IBU-loaded solid dispersions.

As is evident from Figure 4A,B, in the IBU-loaded HPMC solid dispersion, no melting endotherm of IBU was found for any solid dispersions, suggesting the presence of an amorphous drug. The Tg of the systems was evaluated and was found to be 55.0 °C and 50.1 °C in the case of the IBU-HPMC system with 20% and 30% loading, respectively. The GT model predicted a Tg value of 59 °C, while 39 °C was predicted for the systems containing 30% IBU. The IBU-SOL melt-extruded samples demonstrated Tg values of 47.2 °C and 46.3 °C for 20% and 30% loading, respectively. The presence of single Tg values and absence of a melting peak indicates that the interactions between the API and polymer are stronger than drug–drug and polymer–polymer interactions (as suggested by the respective IR data). However, the position of the Tg in the case of SD formulation was difficult to determine, possibly due to the higher heterogeneity. A distinct Tg was observed for the ASDs in certain cases, while in others, a clear Tg could not be identified. Nonetheless, the absence of an API melting point confirms the amorphous state of the formulation.

The mDSC curves of the raw materials are provided in the Supplementary Information sheet (Figures S4, S5 and S8).

##### WAXS

For the 20% IBU-HPMC melt-extruded solid dispersion, no Braggs peak was found; however, in the case of the 30% IBU-HPMC melt-extruded solid dispersion, the characteristic Bragg peak of crystalline/partial crystalline IBU at ~20.16° and 22.33° (Figure S7) was observed (in accordance with the mDSC results). In the case of IBU-SOL solid dispersion prepared via HME and SD, no Bragg peaks of crystalline IBU were observed, indicating presence of amorphous structures of API.

#### 3.1.3. FB-Loaded Solid Dispersion

##### FTIR Spectroscopy

The FTIR spectra of pure crystalline FB shows characteristic IR absorption bands at 2983 and 2935 cm^−1^ (corresponding to benzene ring), 1726 (corresponding to the C=O stretching), 1649 and 1599 (lactone carbonyl functional group), and 1247 cm^−1^ (corresponding to C–O stretching) [30].

**Figure 5:**
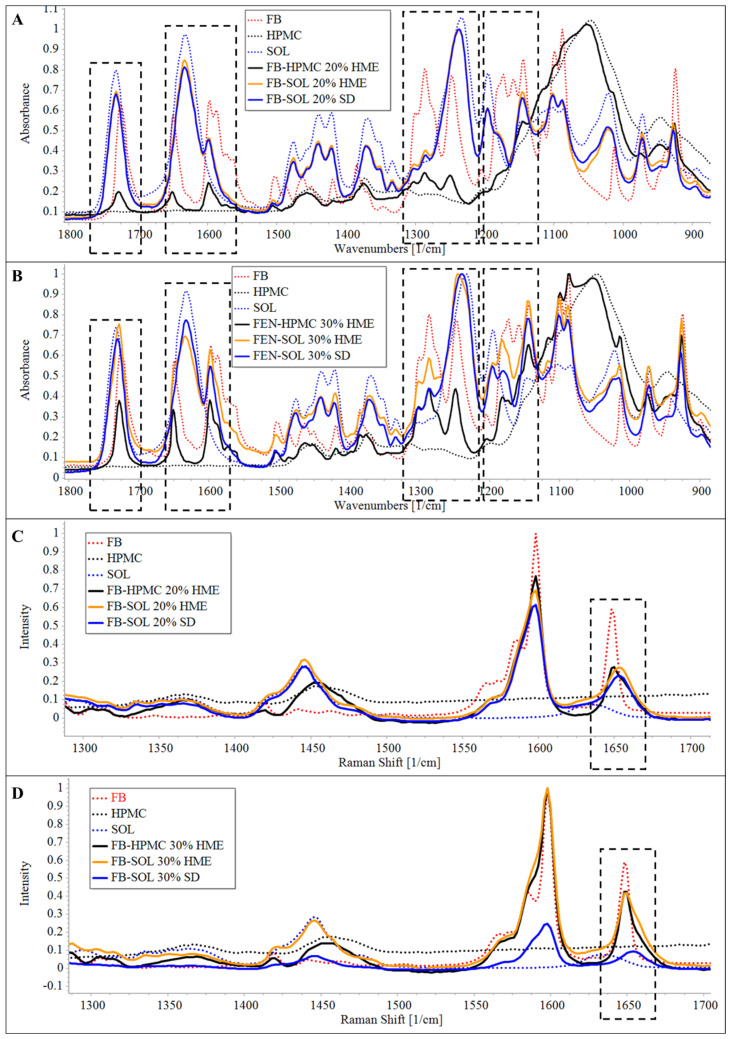
FTIR (**A**,**B**) and Raman spectra (**C**,**D**) of 20% and 30% FB-loaded spray-dried and melt-extruded dispersions, respectively.

Figure 5A,B show the FTIR spectra of the 20 and 30% FB-loaded HPMC- and SOL-based melt-extruded solid dispersions. As evident from the spectra, no significant shift in peak maxima and broadening of signals was observed in the case of the HPMC-based formulations, indicating absence of any interaction between HPMC and FB, required to stabilize the solid dispersion. In the case of the SOL-based formulation, a shift in peak maxima of FB from 1649 to 1633 cm^−1^ and 1247 to 1237 cm^−1^ and a broadening of the peak were observed, suggesting a higher potential of SOL in maintaining an amorphous solid dispersion of FB, as compared to HPMC.

The FTIR spectra of the 20% FB-loaded SOL-based solid dispersion prepared via SD and HME were found to be comparable. For the 30% FB-loaded systems, in the spectra of the solid dispersion, the peak maxima of 1247 cm^−1^ was shifted to 1244 and 1240 cm^−1^ in the case of HME and SD formulations, respectively. Moreover, in the spectral region between 1732 and 1724 cm^−1^ for the HME dispersion with 30% FB content, the peak maximum was at 1728 cm^−1^, while that for the SD dispersion showed a peak maximum at 1732 cm^−1^ (the peak correspondence to the acetate C=O stretching of SOL). Also, the HME dispersion with 30% FB revealed a prominent peak of crystalline FB in the region of 1649 cm^−1^, suggesting the possibility of partial API crystallinity. Thus, as is evident from the spectral data, SD was found to be suitable for the preparation of FB-loaded solid dispersion as compared to the HME method.

##### Raman Spectroscopy

The spectroscopic study of Heinz et al. [31] on the solid-state form of FB reported an interesting Raman shift in the band corresponding to the C=O stretching of benzophenone. In the crystalline form, it was found at 1650 cm^−1^, while in the amorphous form prepared by melting, it shifted to higher wavenumber values (1656 cm^−1^)

The Raman spectrum of pure crystalline FB (Figure 5C,D) demonstrated sharp bands at 1650 cm^−1^ (corresponding to the C=O stretching of the benzophenone), 1598 cm^−1^ and 1585 cm^−1^ (corresponding to in-plane benzene ring stretching), 1146 cm^−1^ (corresponding to C–O stretching of –C–O–Ph) and 1727 cm^−1^ (corresponding to C=O stretching of the ester) [31].

In the case of 20% and 30% HPMC-based melt-extruded solid dispersion, a trivial shift in the 1650 cm^−1^ and 1727 cm^−1^ peaks was observed, suggesting the presence of crystalline structures of FB. For 20% loaded SOL-based melt-extruded solid dispersion, the 1650 cm^−1^ and 1727 cm^−1^ peaks were found to be shifted to 1653 cm^−1^ and 1734 cm^−1^, respectively. However, in the case of the 30% loaded SOL melt-extruded system, the 1650 cm^−1^ and 1727 cm^−1^ peak were found to be comparable with the crystalline FB. Thus, the results demonstrated higher stabilization capacity of amorphous structures of FB in the case of SOL as compared to HPMC.

Moreover, the FB-SOL solid dispersion prepared via SD was also evaluated. As evident from Figure 5C,D, both 20% and 30% drug-loaded samples demonstrated shifts in their 1650 cm^−1^ and 1727 cm^−1^ peaks to 1654 cm^−1^ and 1735 cm^−1^, respectively, suggesting the presence of amorphous FB in SD formulations.

##### mDSC

**Figure 6:**
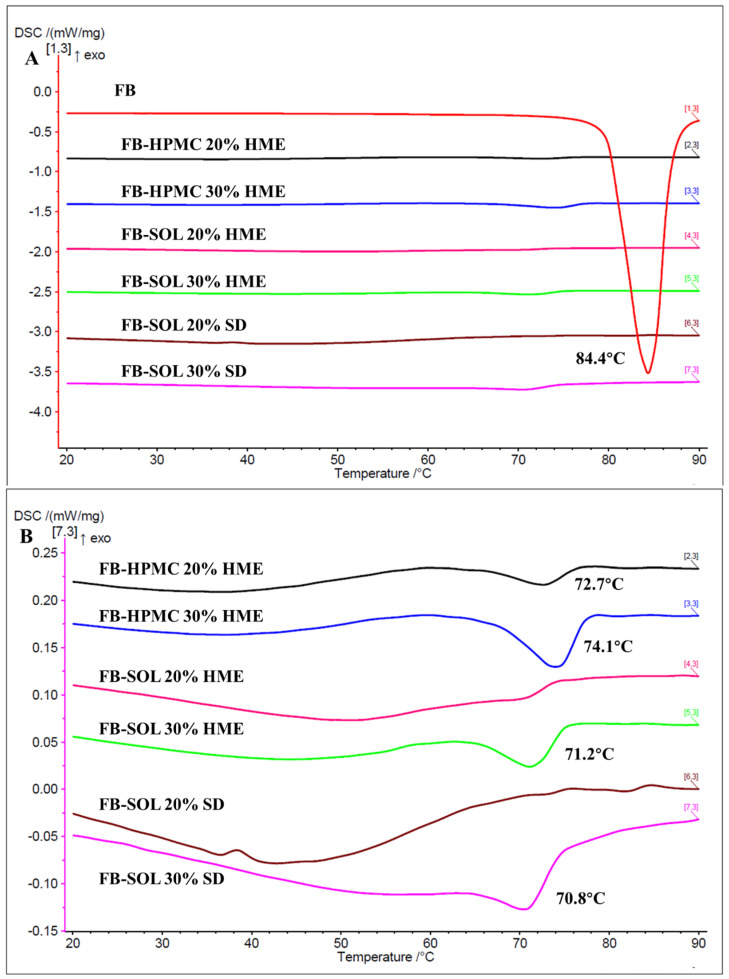
mDSC thermograms of 20% and 30% FB-loaded solid dispersions (**A**) and enlarged image of A (**B**).

As is evident from Figure 6A,B, mDSC thermograms (total heat flow) of 20% and 30% FB-HPMC melt-extruded solid dispersions demonstrated a melting endothermic event at 72.7 °C and 74.1 °C, respectively. The presence of melting peak suggests possible conversion of a fraction of the amorphous FB into fine crystalline nuclei of FB [32]. Moreover, the area of peak (enthalpy) was also evaluated, which was found to be 2.68 and 10.12 J/g for 20% and 30% drug loading, respectively, suggesting higher crystallization in the case of the 30% drug-loaded system.

The mDSC thermograms of the 20% and 30% FB-SOL melt extrudates were also evaluated. The un-milled samples of the FB-SOL solid dispersions exhibited no melting endotherm. However, the cryo-milled samples of the 30% FB-SOL melt extrudate demonstrated a melting peak at 71.2 °C, suggesting the presence of a small fraction of FB crystalline structures. The presence of crystalline structures of FB could be induced via cryo-milling, readily acting as the precursor of API (low Tg) crystallization from the solid dispersion. Furthermore, the enthalpy was also evaluated, which was found to be 5.74 J/g for the 30% loaded solid dispersion. Similar to the 20% FB-SOL HME solid dispersion, the 20% FB-SOL SD solid dispersion demonstrated no melting endotherm. In the 30% loading, a melting endotherm of FB at 70.8 °C was observed, which could be due to the metastable polymorphic form of FB. The results suggest higher potential of SOL as compared to HPMC in maintaining the amorphous state of the API. On the other hand, SD formulations demonstrated comparable stabilizing capacity of amorphous API structures as compared to HME formulations. The mDSC curves of the raw materials are provided in the Supplementary Information sheet (Figures S4, S5 and S11).

##### WAXS

The WAXS profile of the FB-HPMC solid dispersions containing 20% and 30% of drug loading demonstrated characteristic Bragg peaks of crystalline FB, indicating partial crystallization of the FB in the HPMC-based solid dispersions (a representative WAXS pattern of API crystals in solid dispersion is shown in Figure S10). In the case of SOL-based solid dispersions, no distinct Bragg peaks were found for 20% FB-loaded systems prepared via HME and SD. However, for the 30% FB-SOL melt-extruded and spray-dried solid dispersions, residual Bragg peaks of the trace FB crystallites were observed, indicating partial crystallinity of FB in the dispersions. The results were found to be in accordance with the thermal and spectroscopic analysis. A brief summary of the in vitro release study is mentioned in Table 3.

### 3.2. In Vitro Release

The improvement in in vivo solubility and dissolution rate is one of the main drivers towards formulating poorly soluble APIs into amorphous dispersions. Therefore, evaluation of the dispersions prepared via different routes such as SD and HME necessitates the in vitro dissolution studies to access the rate and the extent of the generation of supersaturation and maintenance of the same over the relevant period, besides the improved physical states (molecular miscibility, intermolecular interaction, and amorphicity) and physicochemical stability.

During in vitro release, 10 μm pore size filter papers were used. The selection of this pore size was a deliberate choice aimed at ensuring the accuracy and reliability of our dissolution measurements. Specifically, the 10 μm pore size filters effectively prevent large undissolved particles and aggregates from passing through. Further application of 10 μm pore size filters allowed for us to accurately measure not only the dissolved drug but also the nano-assemblies of the polymer–API complex, with a large hydrodynamic radius. This is crucial for obtaining a true representation of the drug’s dissolution rate and extent from the amorphous solid dispersions (ASDs). Overall, the 10 μm pore size provides a balance between preventing particulate interference and ensuring representative sampling, thereby supporting the integrity and reproducibility of our results.

The release study was conducted at different pH levels for each drug to ensure that the API remained insoluble at those specific pH levels. The pH levels selected for this study were pH 6.8 for CAR, and pH 1.2 for both FB and IBU. These pH values were chosen based on the chemical nature of the drugs: CAR is a weak base, while FB and IBU are weak acids. By conducting this study under conditions where the API is not soluble, it is possible to accurately evaluate the performance and release characteristics of ASDs. This method helps in understanding how ASDs affect the drug release without the interference of the drug dissolving in the testing medium, thereby providing clearer insights into the effectiveness and mechanism of ASDs in drug delivery systems.

As evident from the physiochemical characterization results, the HPMC-based solid dispersions and 30% loaded system demonstrated the presence of a minor fraction of crystalline API. Thus, 20% API-SOL-based solid dispersions prepared via HME and SD were selected for in vitro release study in order to make a comparative dissolution performance assessment.

#### 3.2.1. CAR-Loaded Solid Dispersion

**Figure 7:**
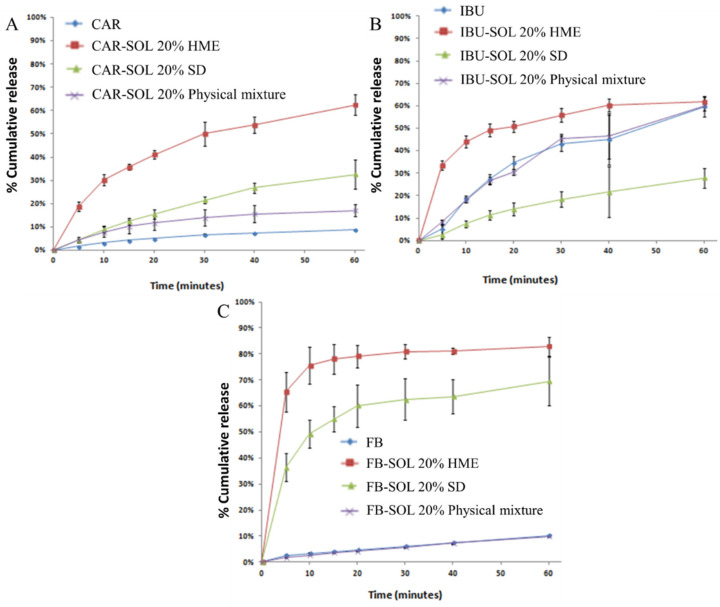
Dissolution profiles of 20% CAR- (**A**), 20% IBU- (**B**) and 20% FB- (**C**) loaded spray-dried and hot melt extrudate solid dispersion.

Dissolution studies on the systems containing CAR were performed using 0.1% *w*/*v* sodium lauryl sulfate (SLS) surfactants and phosphate buffer of pH 6.8. As shown in Figure 7A, the CAR release from crystalline CAR and physical mixture of CAR and SOL within 6 h was found to be 9% and 17%, respectively. The increase in CAR in the physical mixture could be due to the supersaturation capacity of the SOL, as a result of higher interaction between the CAR and SOL (evident from the IR results). Furthermore, a significantly higher rate and extent of CAR release was observed for the CAR-SOL solid dispersions prepared by HME (62.5%) as compared to the SD formulation (32.5%) in 60 min. The increased CAR release from the HME formulation could be due to a predominant increase in wettability and higher surface roughness and larger pores/tortuous structures resulting from cryo-milling.

#### 3.2.2. IBU Loaded Solid Dispersion

The dissolution studies on the samples containing IBU were performed in hydrochloric acid buffer solutions at pH 1.2 with the addition of 0.1% *w*/*v* SLS. As shown in Figure 7B, the rate of release was found to be higher in the case of HME formulation as compared to SD formulations and pure API, which could be due to higher porosity and surface roughness owing to stress during the cryo-milling, paving the way for the rapid migration of solvent into the particles. Interestingly, the release rate of the API in the case of the SD formulation was found to be even lower as compared to physical mixture and pure API. IBU is a strong interacting and low Tg drug; thus, in the case of the SD formulation, the API could have recrystallized during dissolution owing to lower interaction as compared to HME. Thereafter, this possibly facilitated the formation of bigger crystals from the undissolved solid that showed poorer wettability or thus the dissolution compared to the starting crystal form.

#### 3.2.3. FB-Loaded Solid Dispersion

Dissolution studies on the samples containing FB were performed in hydrochloric acid buffer solutions at pH 1.2 with 0.2% *w*/*v* SLS (0.1% *w*/*v* SLS was insufficient to avoid floating). Both solid dispersions evidently showed an improvement in the rate and extent of drug dissolution as compared to the corresponding physical mixture and pure crystalline API (Figure 7C). Overall, the initial rate of dissolution was considerably higher in the case of CAR-SOL dispersions leading to the steady-state within 20 min. The dissolution results demonstrated 83% drug release in the case of HME, significantly higher compared to SD formulations with 70% drug release. Higher release of HME formulation suggested the contribution of various factors effecting the wettability of API, in addition to complete amorphicity of API, for an improved dissolution rate of the drug from polymeric dispersions. However, multi-level microstructures, including that of the polymer inherited from different processing routes, need to be accounted for in the interpretation of such dissolution data.

### 3.3. Stability Studies

As mentioned before, one of the key concerns in ASDs is the physical and chemical stability of the samples during storage. More specifically, moisture and/or thermal stress-induced phase separation and/or crystallization during storage strongly negate the quality attributes of the ASD-based formulations in relation to the solubility advantage. In this work, different ASD systems prepared via two different methods, namely, HME and SD, were assessed to gain insight into the inheritance of (in)stability by the ASDs from their initial physical structures prepared from different processing methods.

#### 3.3.1. Stability Study at 40 °C/75% RH for Three Weeks

##### CAR-SOL Spray-Dried and Melt-Extruded Formulations

- FTIR Spectroscopy

As shown in Figure S13A, the characteristic crystalline peak of CAR at 3342 cm^−1^ was not observed in the case of all the solid dispersions, suggesting amorphous nature of the systems during the stability study. Moreover, the shift in the band attributed to C=O stretching of the caprolactam group of SOL was also found at 1611 cm^−1^. As discussed before, the shift in peak could be attributed to the H-bonding between the carbonyl group of the polymer and API.

- Raman spectroscopy

Raman spectra of all the stability samples of 20% and 30% loaded CAR-SOL were evaluated. Figure S13B demonstrates the peak shift from 1633 cm^−1^ to 1628 cm^−1^ observed, corresponding to the amorphous structure of CAR, indicating presence of amorphous API and stable formulations during storage at 40 °C/75%RH.

- mDSC

**Figure 8:**
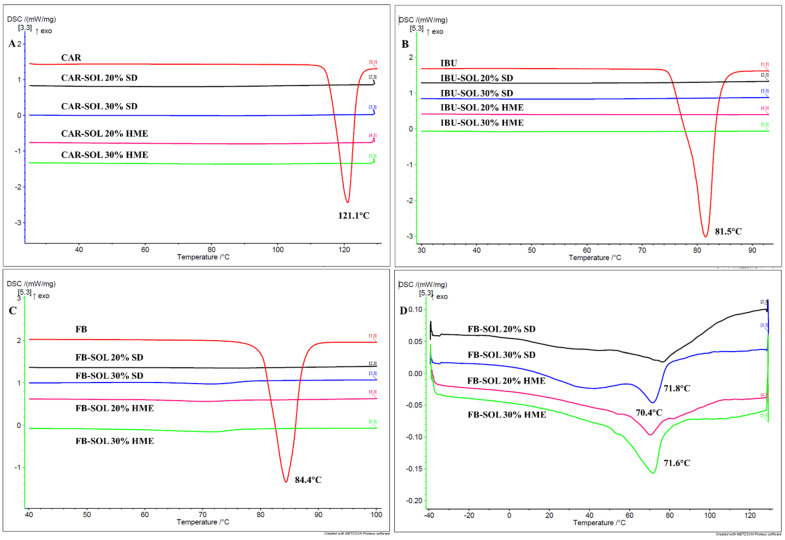
mDSC thermograms of 20% and 30% CAR- (**A**), IBU- (**B**) and FB- (**C**) loaded spray-dried and melt-extruded dispersions (21 days at 40 °C/75%RH), and the enlarged image of C (**D**).

In line with the IR and Raman, an mDSC thermogram of the solid dispersions demonstrated no melting endotherm of the crystalline CAR. The results suggest the presence of API in the amorphous state within the samples during the stability period (Figure 8A).

- WAXS

Wide-angle X-ray scattering patterns of all the dispersions containing CAR and SOL were obtained after 21 days of the stability studies. The results demonstrated no characteristic Bragg peaks in the crystalline structure of CAR after 21 days of incubation at stability conditions (the initial time points, i.e., 7 and 14 days, also exhibited no Bragg peaks. The WAXS pattern of the solid dispersion indicated CAR API to be molecularly dispersed within the polymer matrix in the amorphous state.

##### IBU-SOL Spray-Dried and Melt-Extruded Formulations

- FTIR Spectroscopy

FTIR spectra of all the formulations containing 20% IBU and SOL presented band broadening and a shift in the caprolactam C=O peak from 1633 cm^−1^ to lower wavenumbers (in the range of 1618 cm^−1^–1616 cm^−1^), which could be attributed to the formation of moisture-plasticized caprolactam C=O that are H-bonded with water molecules (Figure S14A). Interestingly, a difference for 30% drug-containing dispersions prepared via a different method was observed. The peak of the caprolactam C=O fraction (1595 cm^−1^) that is H-bonded with IBU molecules showed higher relative intensity than that of free caprolactam C=O (1633 cm^−1^) for HME dispersions. An opposite trend, i.e., higher free caprolactam peak intensity, was observed in comparison to the H-bonded caprolactam, in the case of SD dispersions. Thus, regarding the stability of HME samples, the caprolactam C=O group H-bonded with IBU showed higher intensity compared to that of the caprolactam C=O H-bonded with water, while for SD samples, the intensity of these peaks became equivalent over time. The results suggest lower interaction between SOL and IBU, which could act as a precursor of crystallite formation in the SOL-IBU SD dispersions.

- Raman spectroscopy

After three weeks of stability study, the position of the Raman band attributable to aryl C=C of IBU was observed at 1612 cm^−1^ (amorphous IBU) in the dispersions of IBU and SOL for both 20% and 30% loaded SD and HME solid dispersions (instead of 1608 cm^−1^ for crystalline IBU). This indicates that IBU largely remained amorphous in the formulations until the end of the three weeks of storage at 40 °C/75%RH (Figure S14B).

- mDSC

mDSC thermograms of the HME dispersions containing 20% IBU, during 21 days of stability storage, demonstrated no melting endotherms in the API, indicating the presence of amorphous API (Figure 8B).

- WAXS

All the WAXS patterns of the stability samples of the dispersions showed an absence of the distinct Bragg peaks, indicating that the amorphous nature was maintained during the stability study.

##### FB-SOL Spray-Dried and Melt-Extruded Formulations

- FTIR Spectroscopy

As evident from the FTIR spectra (Figure S15A), the peak attributed to acetate C=O (1732 cm^−1^) and C–O (1230 cm^−1^) stretching of SOL demonstrated slight a redshift and blueshift, respectively, during the stability period. The shift could be attributed to interactions (H-bonding) between the acetate groups of SOL with water molecules (moisture). Moreover, in the case of 20% and 30% drug-loaded solid dispersions (SD and HME), the characteristic peak of crystalline FB at 1650 cm^−1^ was observed after three weeks. In addition, the decrease in the characteristic peak intensity of plasticized caprolactam C=O stretching of SOL (1633 cm^−1^) was also observed, which could be due to decrease in API–carrier interaction. The dominance of the drug peak suggests a possible phase separation and/or crystallization of the API during the stability study. However, the spectral data suggest that the kinetics of the moisture-induced physical instability of the FB-SOL system was found to be higher in the case of the SD dispersion as compared to the melt extrudates.

- Raman spectroscopy

Raman spectra of all the formulations containing FB and SOL (Figure S15B) showed a peak shift in the C=O stretching of the benzophenone group from 1656 cm^−1^ (corresponding to an amorphous structure) to 1650 cm^−1^ (corresponding to a crystalline structure) during the stability period. The peak shift over the storage time suggested progressive crystal growth mediated via high temperature and RH.

- mDSC

As evident from the thermogram, the solid dispersion (with the exception of the stability sample of 20% FB-SOL SD) demonstrated a melting endotherm of FB at approx. 70 °C (Figure 8C). However, in the case of the 30% FB-loaded systems (prepared by HME and SD), comparable enthalpy of FB melting was observed, indicating analogous crystalline conversion of API after 21 days of stability, whereas for the 20% FB-loaded solid dispersions, higher crystallization in the case of hot melt extrudates, compared to spray-dried samples, could be due to small crystals formed during cryo-milling, acting as nuclei.

- WAXS

The WAXS patterns of the HME and SD dispersions (excluding the 20% FB-SOL SD formulations) demonstrated characteristic Bragg peaks of crystalline FB (representative WAXS pattern shown in Figure S12). In line with the spectroscopy and thermal analysis, the WAXS analysis confirms instability in the FB-loaded formulations, irrespective of the method of preparation.

A brief summary of the stability studies is shown in Table 4.

## 4. Discussion

CAR, IBU and FB were selected for this study because they exhibit different propensities to interact with the carrier material and have different Tg. Specifically, CAR is a high-Tg drug, meaning it has a higher glass transition temperature compared to IBU and FB, which are classified as low-Tg drugs. This distinction in Tg is significant, as it influences the thermal and physical stability of the drugs when formulated in ASDs [33,34].

Additionally, CAR and IBU are known to have strong interactions with polymers used in ASDs, which can affect the dissolution rate and bioavailability of the drugs. In contrast, FB exhibits lower interactions with these polymers, which may result in different performance characteristics in the final formulation. By selecting drugs with these varied properties, this study aims to explore the diverse effects of drug–polymer interactions and thermal properties on the development and optimization of amorphous solid dispersions.

HPMC and SOL were selected in our study. These polymers were chosen based on their well-documented efficacy in enhancing the solubility, stability and overall pharmaceutical performance of ASDs [34,35,36]. HPMC is widely recognized for its ability to significantly improve the solubility and dissolution rate of poorly water-soluble drugs, inhibit crystallization, and maintain the amorphous state of drugs over time. Additionally, HPMC’s excellent film-forming properties and biocompatibility make it an ideal candidate for both hot melt extrusion and spray-drying processes.

SOL, on the other hand, is a newer polymer specifically designed for use in solid dispersions, offering unique amphiphilic properties that enhance the solubility and bioavailability of drugs. Its high thermal stability makes it particularly suitable for hot melt extrusion, and its surfactant-like behavior aids in the formulation of stable particles during spray drying. By combining these two polymers, we aimed to leverage their complementary properties and provide a robust comparison of their effects on the solid state, stability and pharmaceutical performance of ASDs. This selection ensures a comprehensive evaluation and broader applicability of our findings across various drug formulation strategies.

### 4.1. CAR-Loaded Solid Dispersions

HME proved to be optimum method for preparing ASDs containing CAR and SOL, in terms of release profile. However, both systems (containing 20 and 30% drug loading) presented high amorphous stability against crystallization of the API. Moreover, the amorphous solid state of the CAR was found to be sustained during the storage at accelerated conditions. The higher stability of the solid dispersion could be due to the high Tg of CAR and possible H-bonding interactions (stronger for SOL). Moreover, the physical stability of the amorphous dispersion of the CAR-SOL and CAR-HPMC systems was maintained under accelerated storage conditions. In spite of the higher Tg of the HPMC system, the non-covalent interactions with CAR were found to be weaker than those observed in CAR-SOL systems.

Dissolution studies showed that CAR-SOL-based ASDs exhibited a marked improvement in dissolution rate compared to pure crystalline API and the corresponding physical mixture. Even though API exists in amorphous form in solid dispersions prepared by SD and HME, the SD-based solid dispersion demonstrated a lower extent and rate of drug release as compared to the HME-based solid dispersion. As evident from the FTIR and Raman analyses, a higher level of interaction was observed between CAR and SOL. This strong interaction likely led to the formation of a robust API–polymer complex. Consequently, this complex formation could be responsible for the slow release of CAR. The strong interactions between the API and the polymer can hinder the drug’s release, as the API is more tightly bound within the polymer matrix, reducing its availability to dissolve and be released into the medium [37].

### 4.2. IBU-Loaded Solid Dispersion

The fresh and stability samples of IBU-SOL ASDs showed the amorphous nature of the API, mainly driven via the high strength of H-bonding between the hydroxyl group of IBU and the caprolactam C=O of SOL. Owing to high interactions, IBU-SOL dispersions partially overcome the hurdle of sub-zero Tg of the API. Moreover, owing to weak interaction between the IBU and HPMC, the IBU-HPMC solid exhibited visual signs of inhomogeneity and instability, which were found to be in accordance with the WAXS results.

The in vitro release profile of the HME-IBU-loaded system depicts a higher rate of dissolution as compared to the free drug and SD formulations. Interestingly, the release rate in the case of the SD formulations was found to be even lower than that of the free drug—which could be due to the higher solubilization of IBU within the SOL and formation of bigger API crystals with limited wettability—during dissolution [37].

### 4.3. FB-Loaded Solid Dispersion

The FB-SOL solid dispersion prepared via HME and SD dispersion with higher drug content demonstrated partial crystallization in fresh samples, owing to the low Tg of FB and a lack of H-donors/acceptors. SD dispersions containing 20% drug loading exhibited higher stability against crystallization compared to HME dispersions. However, the crystallization, in the case of melt extrudates, was mainly driven by the downstream process (cryo-milling) [38]. Thus, different down-streaming of the strands, such as pelletization, direct shaping of the HME strand, congealing, etc., should be screened for. However, in the case of SD, poor yield of the solid dispersion with high drug content was observed. Thus, both HME and SD formulations showed challenges in manufacturing with FB-SOL dispersions.

Moreover, despite partially crystalline content, FB-SOL HME dispersions exhibited higher dissolution rate in comparison to SD dispersions, suggesting the application of various factors, in addition to the solid state of API, responsible for the improved dissolution rate. However, different factors, such as particulate properties, and their correlation with the dissolution performance need to be deeply investigated further.

### 4.4. Overview of Research Work

In this study, we investigated the physicochemical properties and interactions between various APIs and polymers to understand their influence on the stability of ASDs. The results demonstrate that SOL, an amorphous and hydrophilic carrier, exhibits strong hydrogen bond acceptor groups, such as acetate and caprolactam, which enhance intermolecular interactions with APIs. This characteristic is critical in stabilizing the amorphous form of APIs and preventing crystallization. In contrast, HPMC, another amorphous and hydrophilic carrier, has relatively weaker hydrogen bond acceptors, including ether and alcohol groups. This difference in hydrogen bonding capacity influences the degree of stabilization provided to the APIs, often resulting in partial crystallization over time.

Examining the stability against crystallization of the formulations, CAR with its high glass transition temperature (Tg) and –OH functional groups forms strong hydrogen bonds with polymers, particularly SOL. These strong interactions stabilize the amorphous form of CAR, as evidenced by the high stability against crystallization in formulations with SOL. Conversely, formulations with HPMC exhibit weaker interactions, leading to partial crystallization of CAR over time. IBU, which has a sub-zero Tg and a –COOH functional group acting as a strong hydrogen bond donor, ensures robust interactions with polymers. Despite its low Tg, the strong interactions with SOL maintain the amorphous structure and stability of IBU formulations. However, formulations with HPMC show a tendency towards crystallization and inhomogeneity, highlighting the relatively weaker interactions compared to those with SOL. FB, with a sub-zero Tg and no hydrogen bond donors, exhibits lower interactions with the selected polymers, leading to a high crystallization tendency and non-homogeneity in ASDs manufactured with both SOL and HPMC.

Furthermore, the dissolution studies reveal that HME formulations across all three APIs demonstrated the most significant improvement in dissolution rates. This enhancement could be attributed to the cryo-milling effect, which improves wettability and surface roughness, and creates porous/tortuous structures.

In summary, the stability of ASDs is significantly influenced by the strength of hydrogen bonding interactions between APIs and polymers. SOL, with its strong hydrogen bond acceptors, consistently outperforms HPMC in stabilizing APIs and preventing crystallization. These findings underscore the importance of selecting appropriate polymers based on their interaction potential with specific APIs to achieve stable and homogeneous ASD formulations.

## 5. Conclusions and Future Perspectives

The present study investigated the effects of the manufacturing process (HME and SD) and different excipients on the solid-state, physicochemical property, and in vitro performance of ASDs of various APIs. The results revealed that APIs with higher Tg, such as CAR, demonstrated stronger physical interactions with the polymer, leading to greater stability compared to solid dispersions of APIs with lower Tg values. In contrast, manufacturing dispersions of APIs with sub-zero Tg, such as IBU and FB, via HME or SD, resulted in a lack of physical stability against recrystallization. Additionally, SOL exhibited higher interaction with APIs due to its plasticizing effect and the presence of hydrogen donor/acceptor groups compared to HPMC.

This study underscored that Tg and the interaction between the API and polymer are critical factors in maintaining the amorphous state of the API during the shelf-life period. Despite similar physical stability between SD and HME solid dispersions, the in vitro release profiles of HME formulations were significantly higher compared to those of SD formulations. This indicates that particulate properties play a crucial role in determining product performance.

Thus, a rational comparison of ASDs manufactured via different techniques, such as HME and SD, using a broader range of excipients can provide a comprehensive overview of the relationships between material properties, processing methods and product performance. This understanding can expedite the pharmaceutical development of products based on ASDs, ensuring better stability and enhanced drug release profiles.

## Figures and Tables

**Table 1:** Molecular structure and properties such as glass transition temperature (Tg), melting temperature (Tm), true density (ρ), H-bond donors and H-acceptors of APIs and/or carriers.

	Molecular Structure	From the Literature		Experimental Data			Water Solubility	H-Bond Donor	H-Bond Acceptors
		Tg [°C]	Tm [°C]	Tg [°C]	Tm [°C]	ρ [g/cm^3^]			
IBU	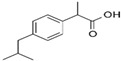	−45.3 ± 1 [12]	75–77.5 [13]	−46.0 ± 1.6	81.5 ± 0.1	1.06	21 mg/L at 25 °C [14]	–OH	–R=O
CAR	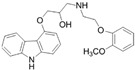	39.58 [15]	118.7 [15]	39.7 ± 0.2	120.9 ± 0.2	1.21	Practically insoluble (0.583 mg/L at 25 °C) [16]	N–H	R–O–R
FB	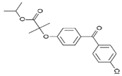	−20.0 [17]	80.5 [18]	−19.5 ± 0.3	84.2 ± 0.1	1.19	0.25 mg/mL at 25 °C [18]	-	R–O–R, –R=O
SOL	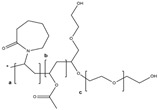	Approx. 70 [19]	-	72.1 ± 3.3	-	1.16	Soluble in water [19]	-	–R=O, R–C(O)–N–R2
HPMC	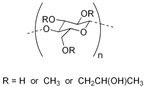	110–120 [20]	-	106.2 ± 3.0	-	1.21		–OH	R–O–CH_3_

**Table 2:** Systems chosen in the present work for manufacturing amorphous solid dispersions via spray drying and hot melt extrusion.

Spray Drying	Hot Melt Extrusion
CAR 20%–SOL 80%	CAR 20%–SOL 80%
CAR 30%–SOL 70%	CAR 30%–SOL 70%
IBU 20%–SOL 80%	IBU 20%–SOL 80%
IBU 30%–SOL 70%	IBU 30%–SOL 70%
FB 20%–SOL 80%	FB 20%–SOL 80%
FB 30%–SOL 70%	FB 30%–SOL 70%
	CAR 20%–HPMC 80%
	CAR 30%–HPMC 70%
	IBU 20%–HPMC 80%
	IBU 30%–HPMC 70%
	FB 20%–HPMC 80%
	FB 30%–HPMC 70%

**Table 3:** Summary of in vitro release studies.

Drug-Loaded Solid Dispersion	pH Conditions	Surfactant	Release Performance in 60 min of In Vitro Release Study
CAR	pH 6.8	0.1% SLS	62.5% and 32.5% for HME and SD formulations, respectively.
IBU	pH 1.2	0.1% SLS	61% and 25% for HME and SD formulations, respectively.
FB	pH 1.2	0.2% SLS	83% and 70% for HME and SD formulations, respectively.

**Table 4:** Summary of stability studies.

Drug System	Stability Condition	Techniques Used	Characterization Methods	Observations
CAR-SOL	40 °C/75% RH, 3 weeks	SD, HME	FTIR, Raman, mDSC, WAXS	No crystalline CAR observed in FTIR, WAXS and mDSC; amorphous nature maintained during stability
IBU-SOL	40 °C/75% RH, 3 weeks	SD, HME	FTIR, Raman, mDSC, WAXS	Amorphous IBU observed in Raman; no crystalline melting observed in mDSC and WAXS; amorphous nature maintained during stability
FB-SOL	40 °C/75% RH, 3 weeks	SD, HME	FTIR, Raman, mDSC, WAXS	Shifts in FTIR and Raman peaks indicating crystalline FB formation; crystalline peaks observed in WAXS

## Data Availability

The original contributions presented in the study are included in the article/Supplementary Materials, further inquiries can be directed to the corresponding author/s.

## Supplementary Materials

The following supporting information can be downloaded at: https://www.mdpi.com/article/10.3390/pharmaceutics16081035/s1, Figure S1: FT-IR spectra of crystalline CAR (a) and amorphous CAR (b) in the region of 4200 and 2400 cm^−1^. Figure S2: Representative WAXS profile of amorphous CAR in HME 30%CAR-HPMC solid dispersion. Figure S3: Modulated differential scanning calorimetry thermograms of pure carvedilol: first heating curve (green) shows melting endotherm peak; second heating curve (purple) shows Tg. Figure S4: Modulated differential scanning calorimetry thermograms of pure Soluplus^®^: first heating curve (green) and second heating curve (purple) show no melting endotherm peak, confirming the amorphous state of the polymer. Figure S5: Modulated differential scanning calorimetry thermograms of pure HPMC: first heating curve (green) second heating curve (purple) show no melting endotherm peak, confirming the amorphous state of the polymer. Figure S6: Zoomed Raman spectra of (a) 20% and (b) 30% CAR loaded spray-dried and melt-extruded dispersions. Figure S7: Representative WAXS profile of crystalline IBU in HME 30%IBU-HPMC solid dispersion. Figure S8: Modulated differential scanning calorimetry thermograms of pure IBU: first heating curve (green) shows melting endotherm peak; second heating curve (purple) shows Tg. Figure S9: Zoomed Raman spectra of (a) 20% and (b) 30% IBU loaded spray-dried and melt-extruded dispersions. Figure S10: Representative WAXS profile of crystalline FB in HME 30%FB-HPMC solid dispersion. Figure S11: Modulated differential scanning calorimetry thermograms of pure fenofibrate: first heating curve (green) shows melting endotherm peak; second heating curve (purple) shows Tg. Figure S12: Representative WAXS profile of crystalline FB in HME 20%FB-SOL solid dispersion after 3 weeks stability study at 40°C/75%RH. Figure S13: FTIR (A) and Raman spectra (B) of 20% and 30% CAR loaded spray-dried and melt-extruded dispersions (21 days at 40°C/75%RH). Figure S14: FTIR (A) and Raman spectra (B) of 20% and 30% IBU loaded spray-dried and melt-extruded dispersions (21 days at 40°C/75%RH). Figure S15: FTIR (A) and Raman spectra (B) of 20% and 30% FB loaded spray-dried and melt-extruded dispersions (21 days at 40°C/75%RH).
