# Supplementary material for: Amorphous Solid Dispersions: Implication of Method of Preparation and Physicochemical Properties of API and Excipients
Source: Pharmaceutics. 2024 Aug 2;16(8):1035. doi: 10.3390/pharmaceutics16081035 (PMC11360349; doi:10.3390/pharmaceutics16081035)
Supplement: Supplementary file 1 [file pharmaceutics-16-01035-s001.zip › pharmaceutics-3098002-supplementary.pdf]

Amorphous Solid Dispersions: Implication of Method of Preparation and Physicochemical Properties of API and Excipients

Varun Kushwah 1,*, Cecilia Succhielli 1, Isha Saraf 1 and Amrit Paudel 1,2,*

[1] Research Center Pharmaceutical Engineering GmbH, 8010 Graz, Austria

[2] Institute of Pharmaceutical Science, Department of Pharmaceutical Technology, University of Graz, 8010 Graz, Austria

* Corresponding author: Varun Kushwah, varun.kushwah@rcpe.at; Amrit Paudel, amrit.paudel@rcpe.at

Supplementary information

1 HPLC analysis:

1.1 Ibuprofen:

Quantification of Ibuprofen in dissolution samples was performed by HPLC-UV using an Agilent 1260 series Infinity HPLC system (Agilent, Waldbronn, Germany) equipped with a photo diode array detector. A Hibar Purospher® STAR RP-18e (150x4.6 mm, 5µm) column (Merck Millipore, Darmstadt, Germany) was used as stationary phase. Analysis was carried out under isocratic elution conditions using a mobile phase composed of acetonitrile (Sigma Aldrich)/ water (chromatography quality)/ 8.5 wt.% phosphoric acid (HPLC grade, VWR international), 67:32.5:0.5 *v/v* at a flow rate of 1mL/ min. Column temperature was set to 20 °C and an aliquot of 20µL of sample solution was injected into the HPLC system. UV-detection was performed at 231 nm over the run time of 7 minutes. A 9-point external standard calibration curve in the range from 1 µg/mL to 43 µg/mL was prepared for quantification.

1.2 Carvedilol

Quantification of Carvedilol in dissolution samples was performed by UPLC-UV using an Acquity UPLC® H-class system (Waters, Milford, USA) equipped with a photo diode array detector. A waters Acquity UPLC CSH C18 column (75x2.1 mm, 1.7µm) (Waters, Milford, USA) was used as stationary phase. Analysis was carried out under isocratic elution conditions using a mobile phase composed of acetonitrile (Sigma Aldrich)/ aqueous potassium dihydrogen phosphate solution pH 2.0 (1.77 g potassium dihydrogen phosphate in 650 mL water; pH 2.0 adjusted with phosphoric acid 85wt.%), 35:65 *v/v* at a flow rate of 1mL/ min. Column temperature was set to 50 °C and an aliquot of 2µL of sample solution was injected into the HPLC system. UV-detection was performed at 240 nm over the run time of 5 minutes. A 9-point external standard calibration curve in the range from 0.5 µg/mL to 19.0 µg/mL was prepared for quantification.

1.3 Fenofibrate

Quantification of Fenofibrate in dissolution samples was performed by HPLC-UV using an Agilent 1260 series Infinity HPLC system (Agilent, Waldbronn, Germany) equipped with a photo diode array detector. A Waters Spherisorb ODS2 (150x4.6 mm, 3µm) column (Waters, Milford, USA) was used as stationary phase. Analysis was carried out under isocratic elution conditions using a mobile phase composed of acetonitrile (Sigma Aldrich)/ water adjusted with phosphoric acid to pH 2.5, 80:20 *v/v* at a flow rate of 1mL/ min. Column temperature was set to 40 °C and an aliquot of 40µL of sample solution was injected into the HPLC system. UV-

detection was performed at 286 nm over the run time of 6 minutes. A 10-point external standard calibration curve in the range from 0.5 µg/mL to 34.0 µg/mL was prepared for quantification.

2 CAR loaded solid dispersion

2.1 FTIR spectroscopy

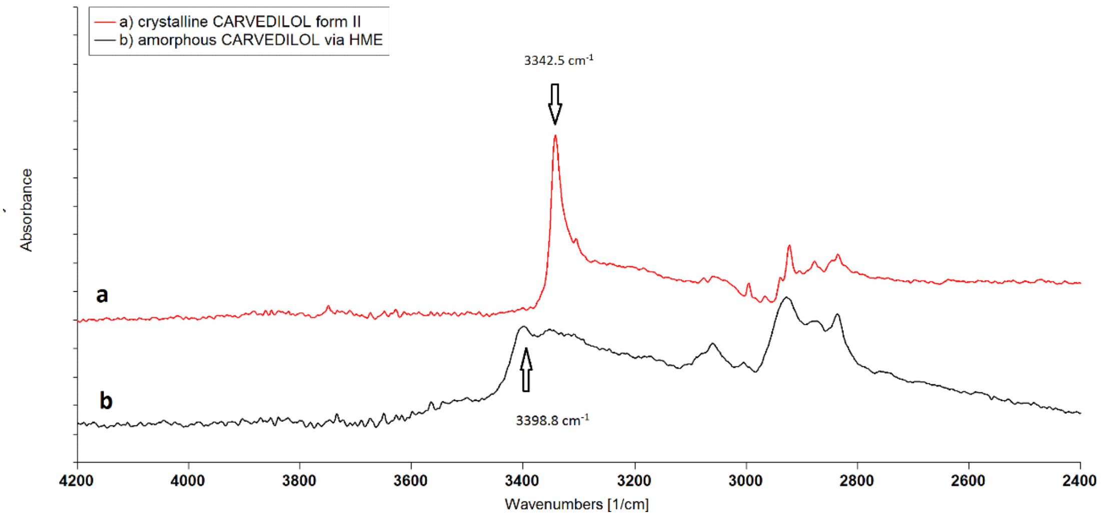

Figure S1: FT-IR spectra of crystalline CAR (a) and amorphous CAR (b) in the region of 4200 and 2400 cm-1.

2.2 WAXS analysis

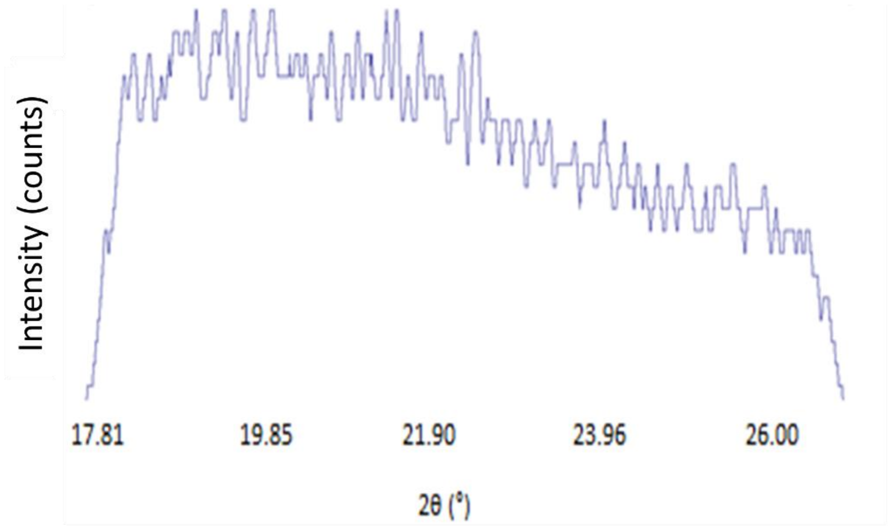

Figure S2: Representative WAXS profile of amorphous CAR in HME 30%CAR-HPMC solid dispersion

2.3 mDSC analysis

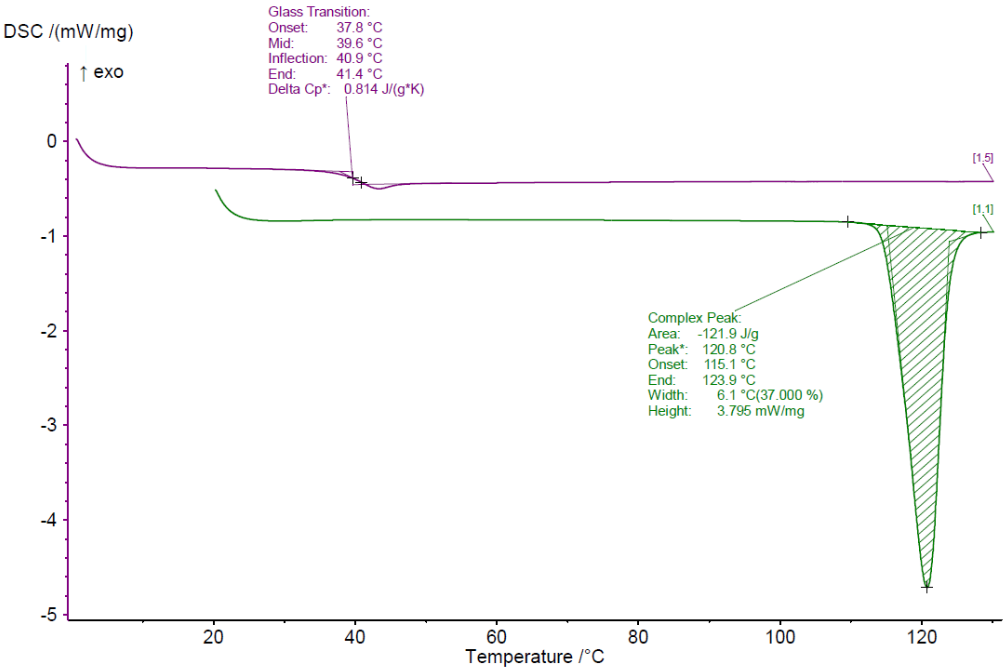

Figure S3: Modulated differential scanning calorimetry thermograms of pure carvedilol: first heating curve (green) shows melting endotherm peak; second heating curve (purple) shows Tg.

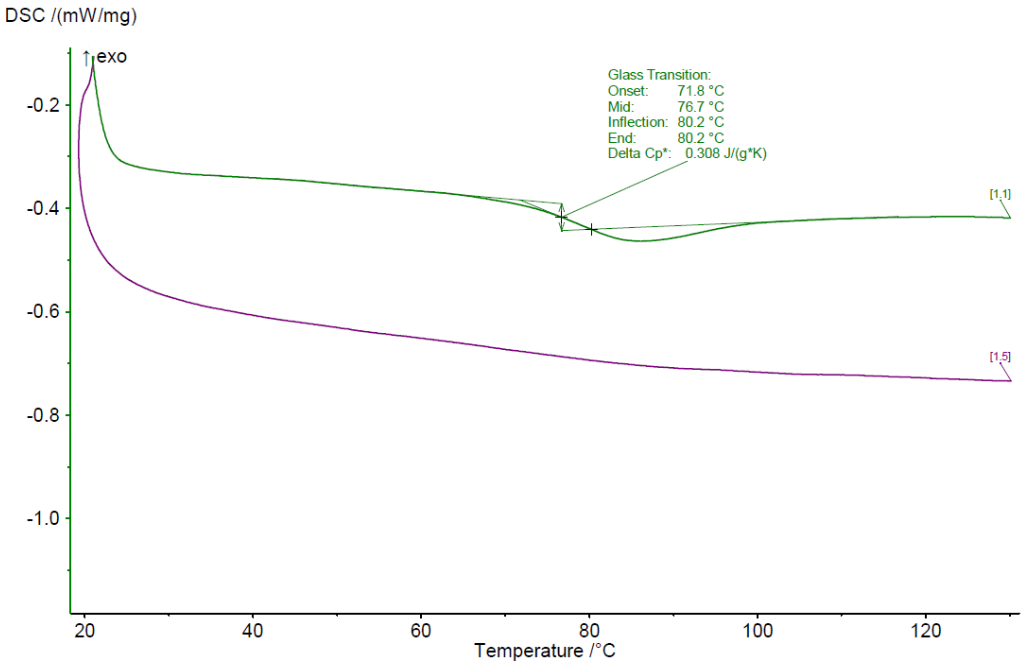

Figure S4: Modulated differential scanning calorimetry thermograms of pure Soluplus®: first heating curve (green) and second heating curve (purple) show no melting endotherm peak, confirming the amorphous state of the polymer.

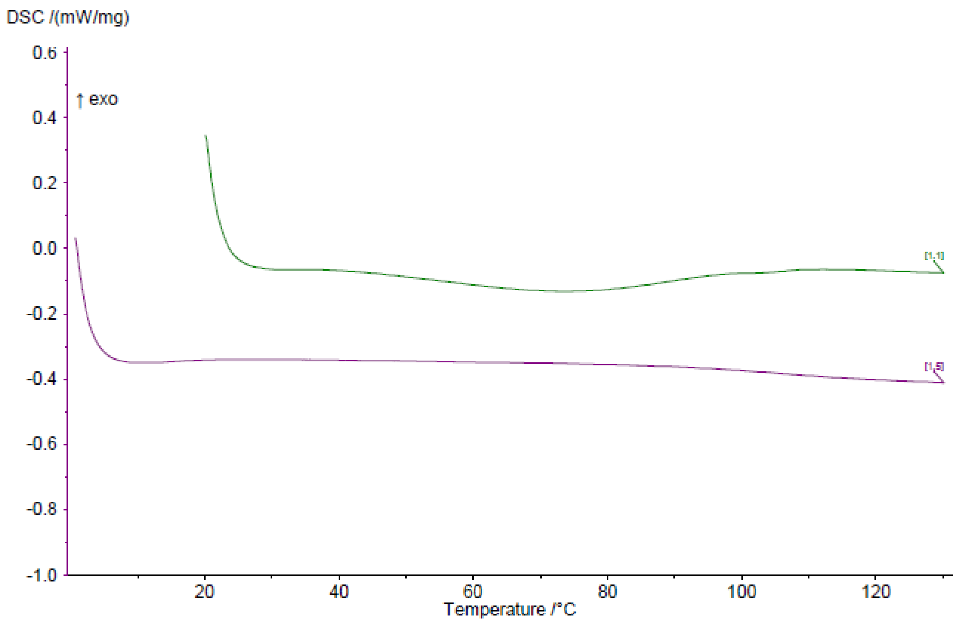

Figure S5: Modulated differential scanning calorimetry thermograms of pure HPMC: first heating curve (green) second heating curve (purple) show no melting endotherm peak, confirming the amorphous state of the polymer.

2.4 Raman analysis

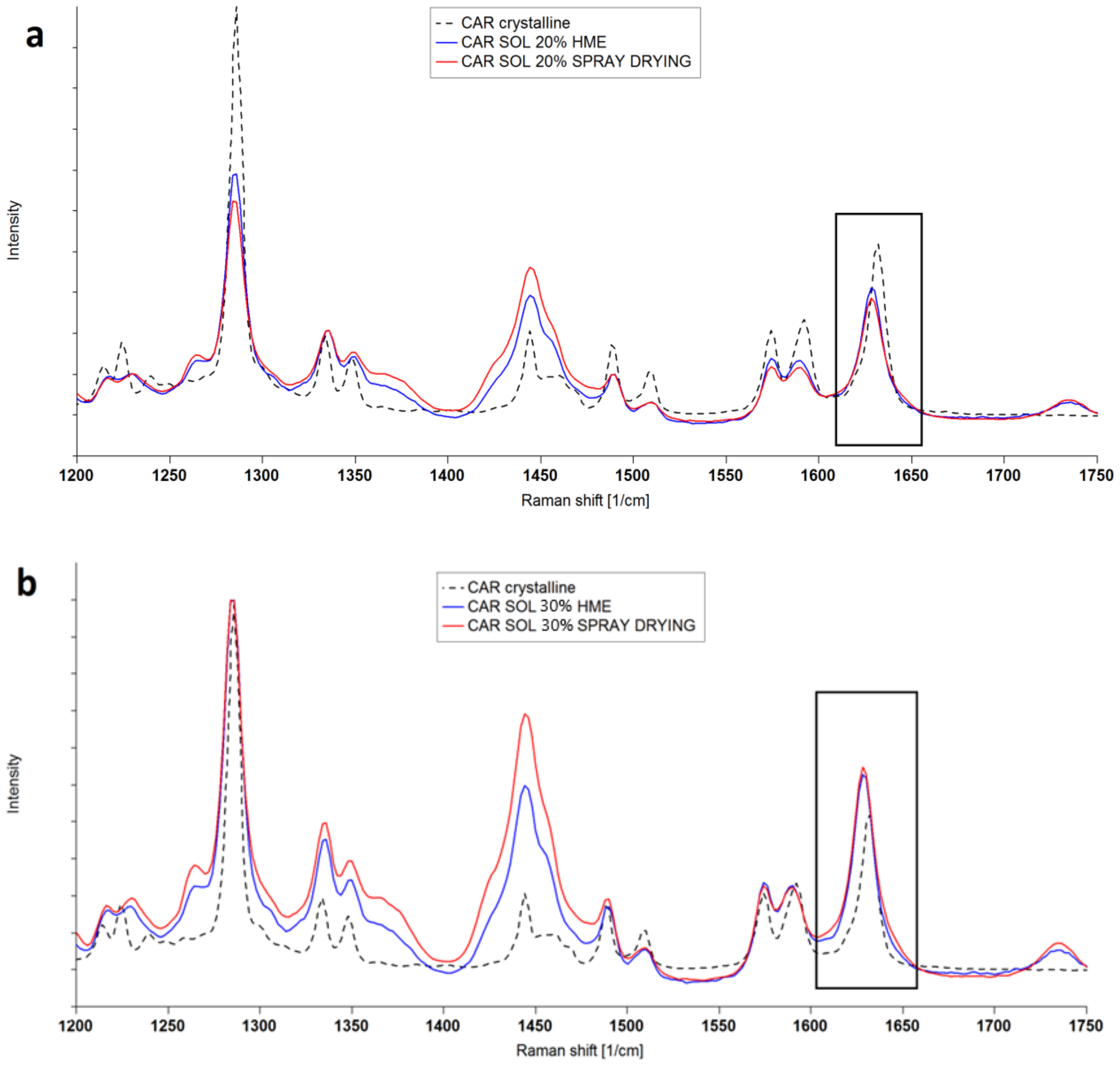

Figure S6: Zoomed Raman spectra of (a) 20% and (b) 30% CAR loaded spray-dried and melt-extruded dispersions

3 IBU loaded solid dispersion

3.1 WAXS analysis

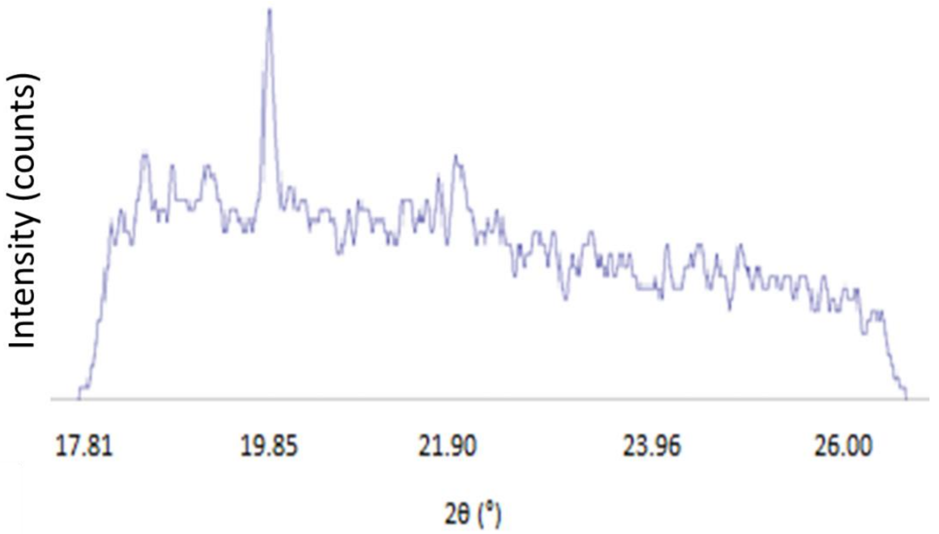

Figure S7: Representative WAXS profile of crystalline IBU in HME 30%IBU-HPMC solid dispersion

3.2 mDSC analysis

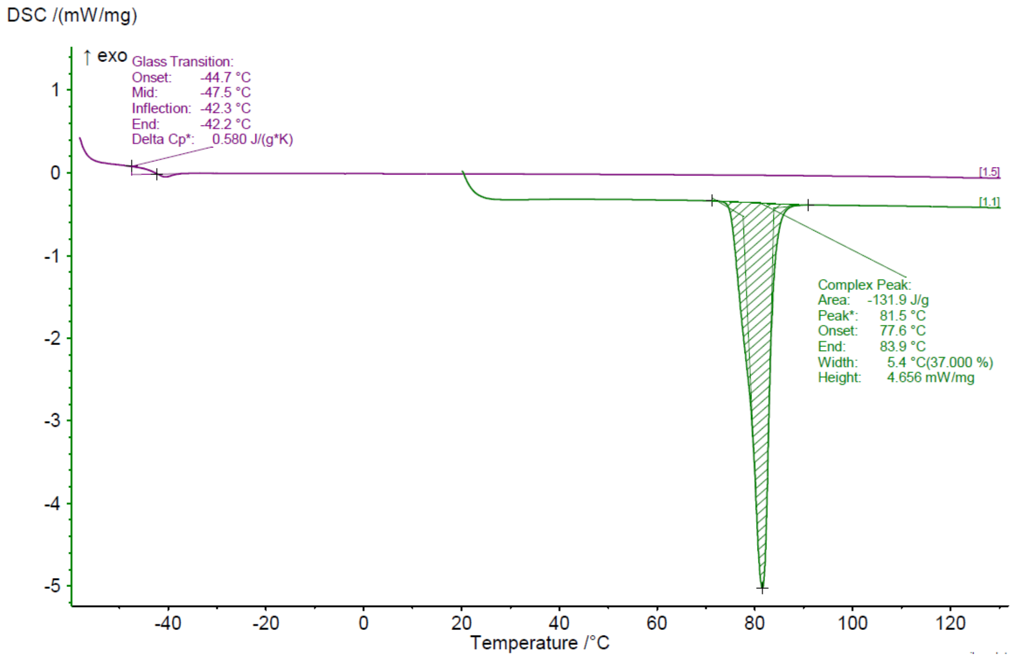

Figure S8: Modulated differential scanning calorimetry thermograms of pure IBU: first heating curve (green) shows melting endotherm peak; second heating curve (purple) shows Tg.

3.3 Raman analysis

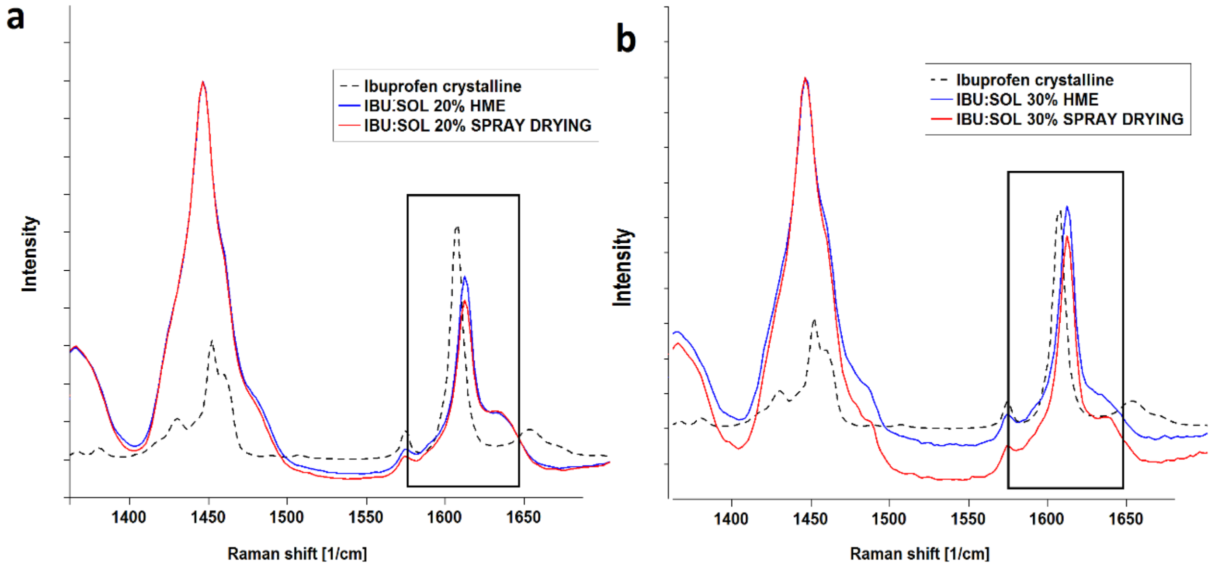

Figure S9: Zoomed Raman spectra of (a) 20% and (b) 30% IBU loaded spray-dried and melt-extruded dispersions

4 FB loaded solid dispersion

4.1 WAXS analysis

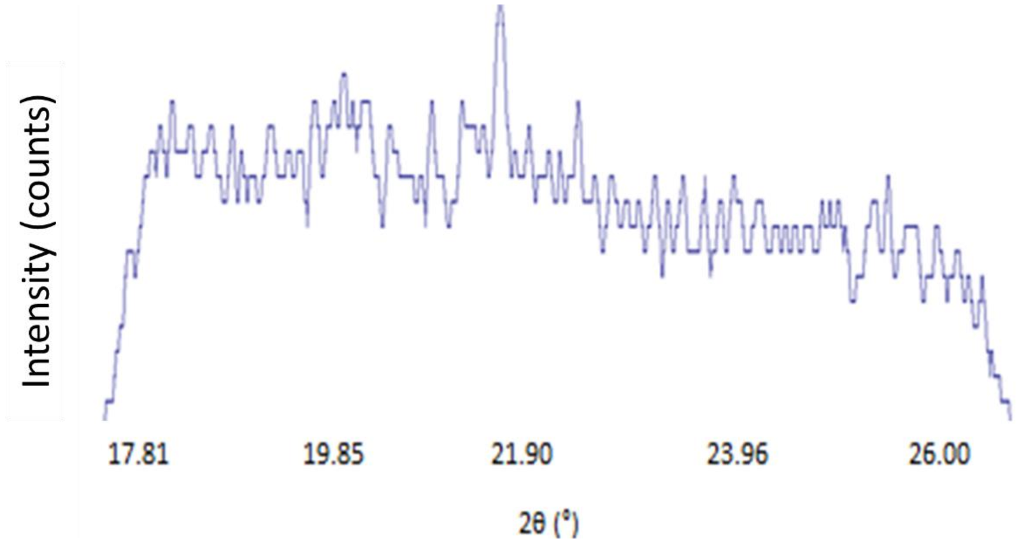

Figure S10: Representative WAXS profile of crystalline FB in HME 30%FB-HPMC solid dispersion

4.2 mDSC analysis

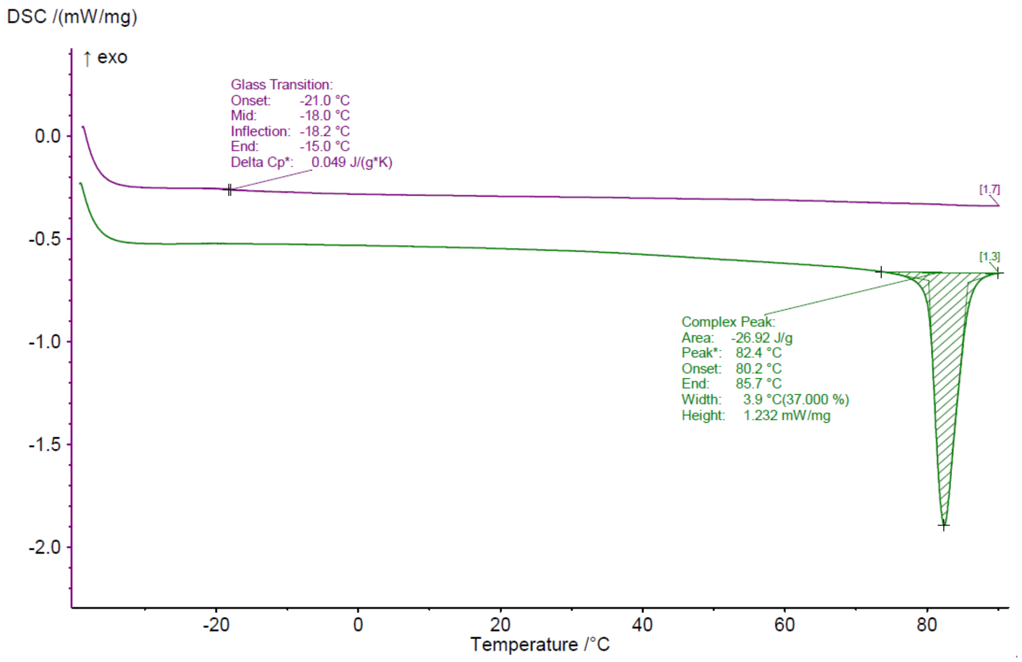

Figure S11: Modulated differential scanning calorimetry thermograms of pure fenofibrate: first heating curve (green) shows melting endotherm peak; second heating curve (purple) shows Tg.

5 Stability study

5.1 WAXS analysis of FB loaded solid dispersion

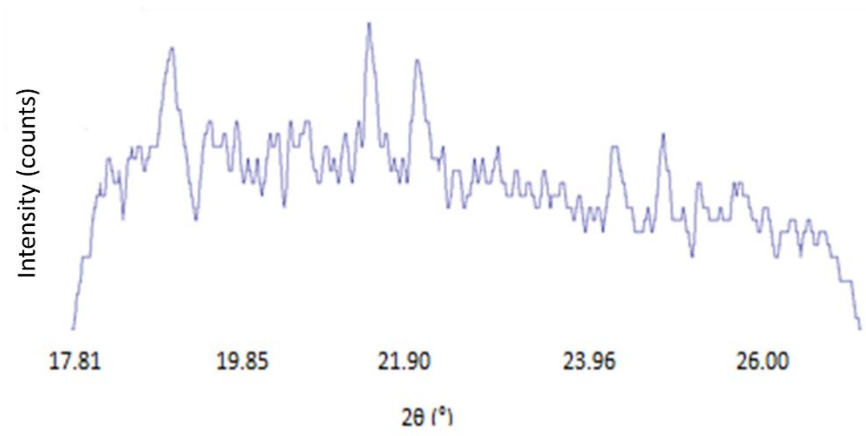

Figure S12: Representative WAXS profile of crystalline FB in HME 20%FB-SOL solid dispersion after 3 weeks stability study at 40°C/75%RH.

5.2 Raman and FTIR analysis of stability samples

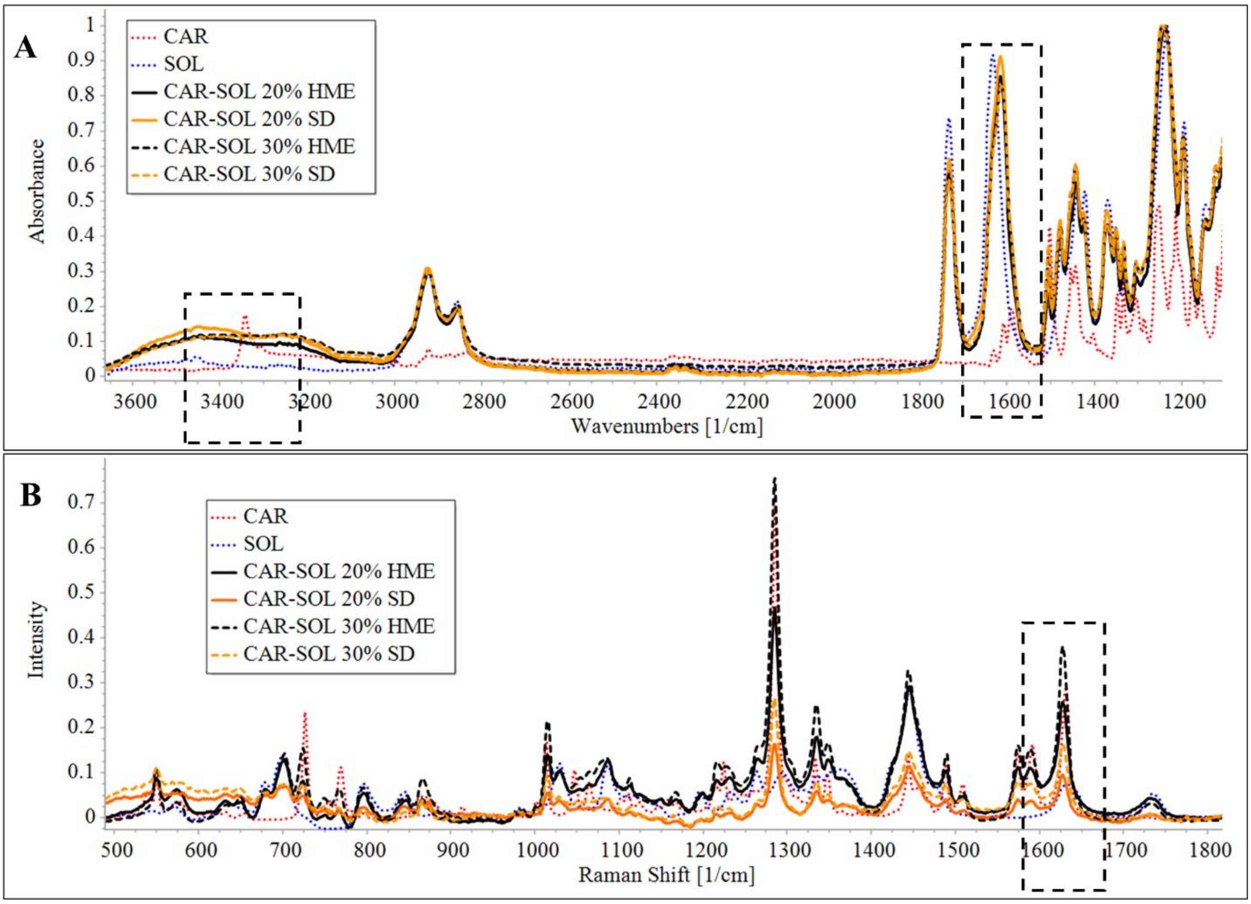

Figure S13: FTIR (A) and Raman spectra (B) of 20% and 30% CAR loaded spray-dried and melt-extruded dispersions (21 days at 40°C/75%RH).

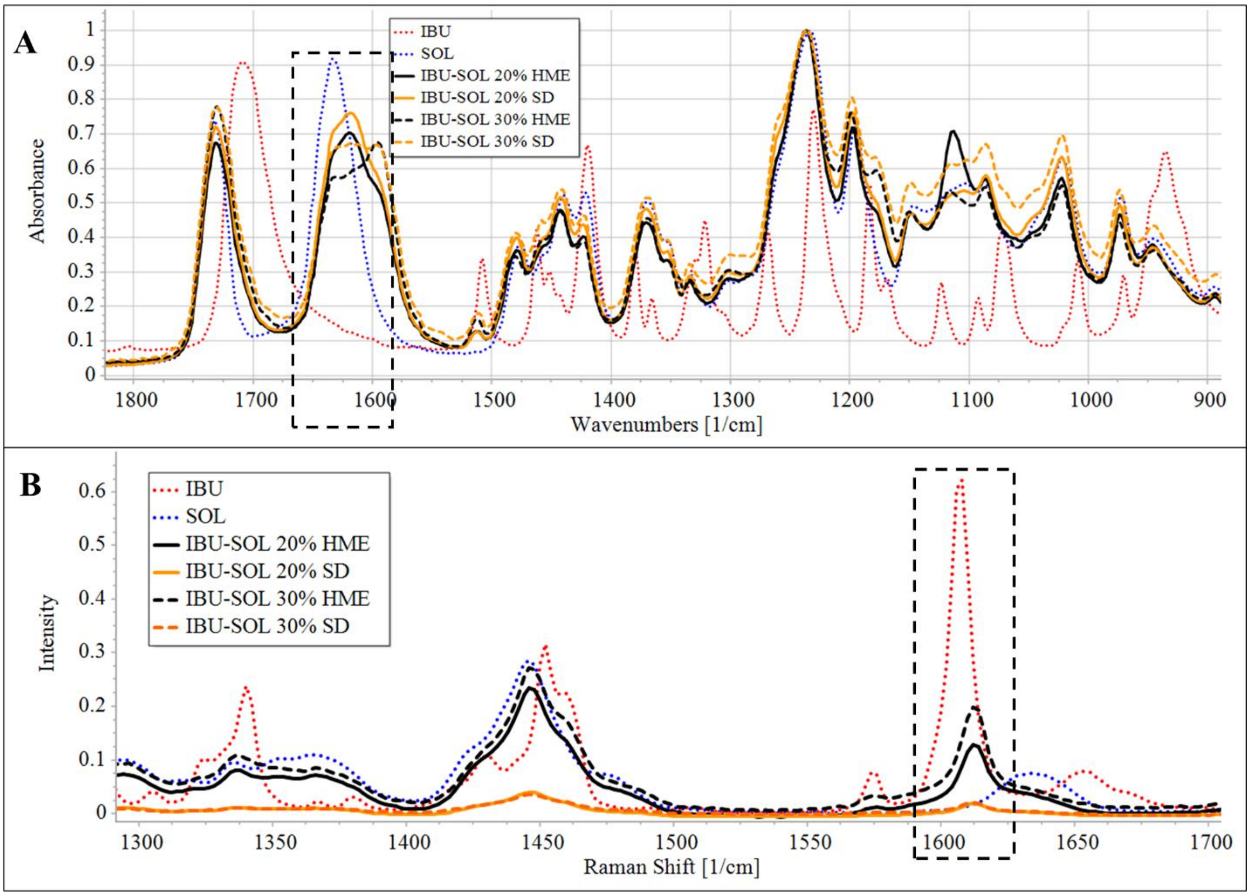

Figure S14: FTIR (A) and Raman spectra (B) of 20% and 30% IBU loaded spray-dried and melt-extruded dispersions (21 days at 40°C/75%RH).

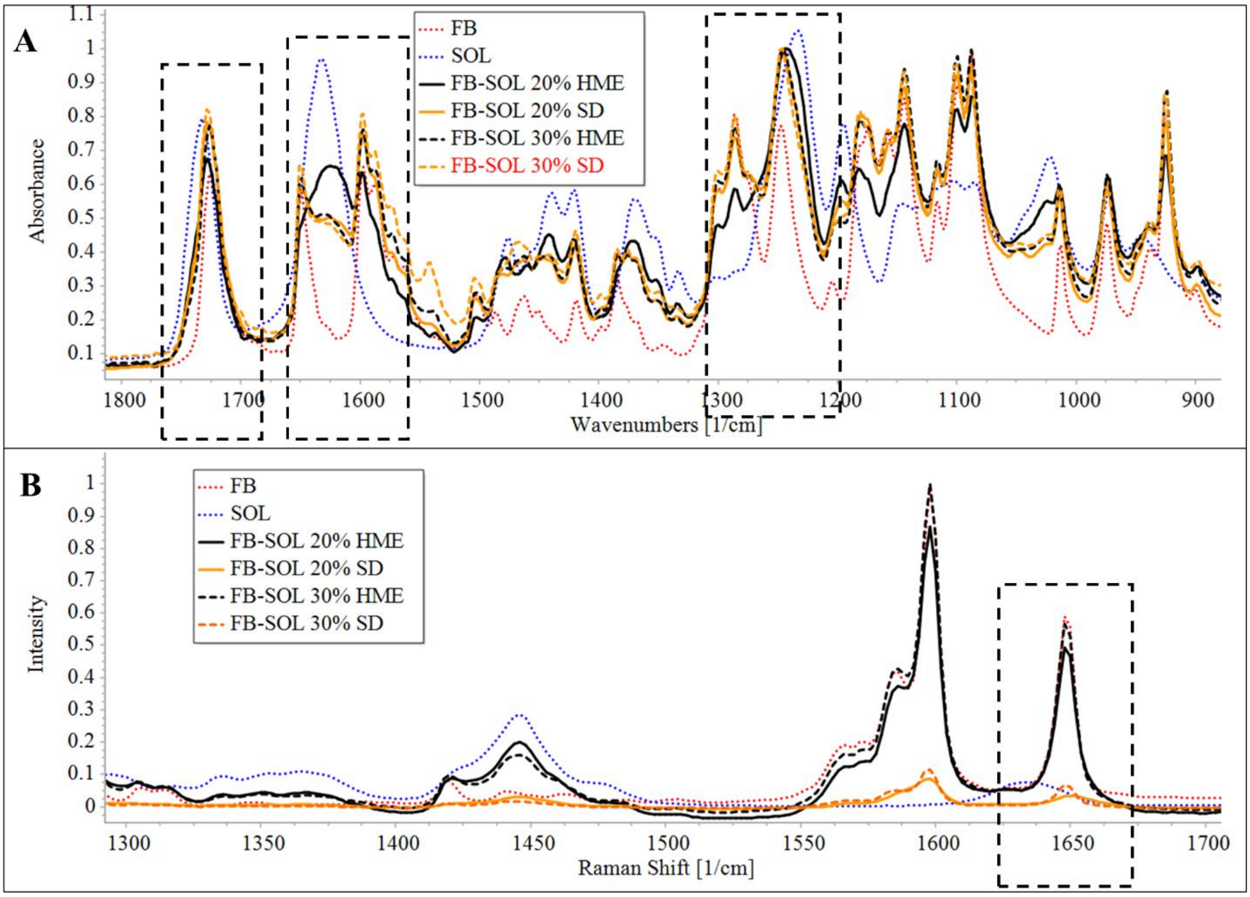

Figure S15: FTIR (A) and Raman spectra (B) of 20% and 30% FB loaded spray-dried and melt-extruded dispersions (21 days at 40°C/75%RH).
